# Alteration of β-glucan in the emerging fungal pathogen *Candida auris* leads to immune evasion and increased virulence

**DOI:** 10.1007/s00430-024-00795-y

**Published:** 2024-07-05

**Authors:** Shiela Marie Gines Selisana, Xinyue Chen, Eny Mahfudhoh, Anom Bowolaksono, Anna Rozaliyani, Kanami Orihara, Susumu Kajiwara

**Affiliations:** 1 School of Life Science and Technology, Tokyo Institute of Technology, 4259 Nagatsuta-cho, Midori-ku, Yokohama, Kanagawa 226-8501 Japan; 2 Department of Biology, Faculty of Mathematics and Science, University of Indonesia, Depok, 16424 Indonesia; 3 Faculty of Medicine, University of Indonesia, Jakarta, 10430 Indonesia

**Keywords:** *Candida auris*, β-glucan, Cell wall remodeling, Phagocytosis, Silkworm

## Abstract

*Candida auris* is an emerging pathogenic yeast that has been categorized as a global public health threat and a critical priority among fungal pathogens. Despite this, the immune response against *C. auris* infection is still not well understood. Hosts fight *Candida* infections through the immune system that recognizes pathogen-associated molecular patterns such as β-glucan, mannan, and chitin on the fungal cell wall. In this study, levels of β-glucan and mannan exposures in *C. auris* grown under different physiologically relevant stimuli were quantified by flow cytometry-based analysis. Lactate, hypoxia, and sublethal concentration of fluconazole trigger a decrease in surface β-glucan while low pH triggers an increase in β-glucan. There is no inverse pattern between exposure levels of β-glucan and mannan in the cell wall architecture among the three clades. To determine the effect of cell wall remodeling on the immune response, a phagocytosis assay was performed, followed by quantification of released cytokines by ELISA. Lactate-induced decrease in β-glucan leads to reduced uptake of *C. auris* by PMA-differentiated THP-1 and RAW 264.7 macrophages. Furthermore, reduced production of CCL3/MIP-1⍺ but not TNF-⍺ and IL-10 were observed. An in vivo infection analysis using silkworms reveals that a reduction in β-glucan triggers an increase in the virulence of *C. auris*. This study demonstrates that β-glucan alteration occurs in *C. auris* and serves as an escape mechanism from immune cells leading to increased virulence.

**Supplementary Information:**

The online version contains supplementary material available at 10.1007/s00430-024-00795-y.

## Introduction

*Candida* is the largest genus of medically important yeasts and is the most common cause of fungal infections worldwide [1]. Considered part of the normal human microbiome, *Candida* is a commensal microorganism in the skin and mucous membranes of the respiratory, gastrointestinal, and female genital tract of healthy persons. However, in immunocompromised individuals, some species can invade mucosal surfaces and cause mucosal or systemic candidiasis [2]. Candidiasis is one of the most common opportunistic fungal infections and systemic candidiasis is the fourth leading cause of nosocomial bloodstream infections, causing serious infections that can affect the blood, heart, brain, eyes, bones, and other parts of the body [2, 3]. The most frequent cause of candidiasis is *C. albicans* [3]. However, the incidence of candidiasis caused by other species such as the emerging pathogen, *C. auris*, has increased drastically over the years [4]. *C. auris* was discovered in 2009 in the external ear canal of a patient in Japan [5]. Despite its relatively recent discovery, this fungus has been designated as a global public health threat [6] and is categorized as one of the four critical priority fungal pathogens by the World Health Organization (WHO) [7]. *C. auris* causes hospital outbreaks and is difficult to eliminate from hospital intensive care wards due to its resistance to commonly used cleaning agents. Even though *C. albicans* has always been considered the top causal agent of candidiasis, *C. auris* now accounts for nearly 20% of *Candida* bloodstream isolates, surpassing that of *C. albicans* [8]. Most *C. auris* isolates show high levels of resistance to different types of antifungal drugs such as azoles, polyenes, and flucytosine [4]. Furthermore, in vitro studies have shown that more than 90% of *C. auris* isolates are resistant to the antifungal prophylactic drug fluconazole [4].

Fungal cell walls not only provide cell rigidity, shape, and protection from environmental stressors but also allow for immune cell recognition of the hosts. The cell wall contains relevant pathogen-associated molecular patterns (PAMPs) and epitopes for the host immune response. The three most common PAMPs in *Candida* species are β-glucan, chitin, and mannan [9]. These PAMPs are then recognized by pattern recognition receptors (PRRs) found on the surface of human immune cells such as monocytes, macrophages, neutrophils, and dendritic cells [10]. For fungal cell sensing of host cells, the major factor involved is the recognition of β-glucan by dectin-1. This recognition transduces intracellular signaling through several pathways thereby triggering or regulating a wide range of cellular responses such as reactive oxygen species production and cytokine synthesis. Several studies demonstrated that polymorphisms that impair dectin-1 activity in humans are linked to aberrant cytokine responses, as well as a higher risk of recurrent mucocutaneous candidiasis and gut colonization [11]. Other macrophage PRRs are the C-type lectin receptors such as dectin-2, mannose receptor, and dendritic-cell-specific intercellular adhesion molecule-3-grabbing non-integrin (DC-SIGN) that recognize mannan. Moreover, the Toll-like receptors (TLRs) such as TLR2 and TLR4 also recognize mannan-containing structures on the cell wall [11].

Recent studies have shown that *C. albicans* mask its β-glucan when exposed to environmental stimuli such as low oxygen concentration, lack of iron, and high lactate concentrations [12–14]. This poses a threat since masking of a major PAMP could lead to immune evasion, thereby allowing the pathogen to proliferate in the host. To date, the majority of PAMP masking studies have focused on *C. albicans.* Infections caused by non-albicans *Candida* species including *C. auris* increased globally but studies relating to the alteration of β-glucan on these species have not been reported yet. Therefore, considering the medical relevance of *C. auris* and the critical priority level conferred by the Centers for Disease Control and Prevention (CDC) [15] and WHO [7], we determined if physiologically relevant culture conditions trigger alteration of β-glucan in *C. auris*, and if this will affect the in vitro and in vivo host response using human monocyte cell line THP-1, murine macrophage cell line RAW 264.7, and domestic silkworm (*Bombyx mori*).

A thorough understanding of host-pathogen interaction is indispensable. Understanding the relationship between environmental sensing and modulation of the host-*C. auris* pathogen interaction provides new opportunities for the development of possible innovative antifungal strategies.

## Materials and methods

### Fungal strains used in this study

A clinical isolate of *C. auris* from Indonesia (UI001) and type strains ATCC MYA-5001 (B11220) and ATCC MYA-5002 (B11221) were used in this study. For the silkworm infection assay, *C. albicans* TUA6 from the National Institute of Infectious Diseases in Japan and *Saccharomyces cerevisiae* S288C from the Institute of Fermentation in Japan were used. All strains were stored as frozen stocks in 30% glycerol at -80 °C and subcultured on YPD (1% yeast extract, 2% peptone, 2% glucose, 2% agar) plates.

### Surface staining of β-glucan

To determine the effect of certain culture conditions, *C. auris* was grown for 5 h at 37 °C, and the following procedure was conducted. For the effect of an alternative carbon source, cells were grown in a Minimal medium (0.67% yeast nitrogen base with ammonium sulfate) with 2% glucose (control) and varying concentrations of lactate (Nacalai Tesque, Japan) and glycerol (Nacalai Tesque, Japan). For the effect of oxygen level, cells were either grown in hypoxic conditions (< 0.1% O_2_) using a hypoxia culture kit (Sugiyama-gen, Japan), poor aeration condition (without shaking) or normoxic condition (with shaking at 200 rpm). Nitrogen gas (Kenis, Japan) was injected before sealing the hypoxia kit to accelerate oxygen removal. For the effect of pH, different sets of YPD with 3.57% HEPES were prepared at varying pH (4.0, 5.5, 7.0, and 8.5). Cells grown in pH 7 serve as the control. For the effect of antifungal drugs, cells were grown with or without fluconazole (2–16 µg/mL; LKT Laboratories, USA), 5-fluorocytosine (16–500 ng/mL; Tokyo Chemical Industry Co., Ltd, Japan), amphotericin B (31–188 ng/mL; Fujifilm Wako Pure Chemical Corporation, Japan), and micafungin (1–40 µg/mL; Astellas, Japan).

Cells were analyzed following a protocol from a previous study [14] but with slight modifications. Cells were stained with 2.5 ng/µL Fc-hDectin-1a (cat. # fc-hdec1a, InvivoGen, USA) in FACS buffer (1x PBS, 50 mM EDTA, 5% FBS) for 1 h. Cells were washed twice and stained with 1:500 Alexa Fluor 488-conjugated AffiniPure Goat Anti-Human IgG_1_ Fc_γ_ Fragment Specific (code # 109-545-098, Jackson ImmunoResearch Laboratories, Inc., USA) in the dark for 1 h. Cells were washed twice and fixed with 4% *p*-formaldehyde for 15 min. Controls such as unstained cells and cells stained with Alexa Fluor 488 only were used. For confocal microscopy observation, stained cell suspension was observed using LSM 780 upright confocal microscope (Zeiss, Japan). EC800 Flow Cytometry Analyzer (Sony Biotechnology, Inc., Japan) with 488-nm laser in combination with FL1 (525/50) bandpass filter was used to analyze at least 100,000 cells from each sample. The gating strategy was kept constant in all analyses. To generate the histograms, FlowJo v10.8 Software (BD Life Sciences) was used. The fold change is calculated as the mean of the median fluorescence intensities (MFI) of β-glucan::Alexa Fluor 488 in the experimental variable divided by the mean MFI of the control.

### Surface staining of mannan

*Candida* cells were cultured as described previously. Cells were stained with 50 µg/mL concanavalin A-tetramethylrhodamine (cat. # C860, Thermo Fisher Scientific Inc., 2 mg/mL stock in 0.1 M sodium bicarbonate) for 30 min and fixed with 4% *p*-formaldehyde. Unstained cells were used as a control. Flow cytometry and confocal microscope imaging were performed as previously described, but 488 nm and 561 nm lasers in combination with the FL2 (585/40) bandpass filter were used to analyze exposure levels of mannan. The fold change is computed as the mean of the MFI of mannan::TRITC in the experimental variable divided by the mean MFI of the control.

### Phagocytosis assay using THP-1

Fungal cells were collected and stained with 10 µg/mL acridine orange (AO; Sigma Aldrich) for 20 min. For the phorbol 12-myristate 13-acetate (PMA) differentiation of THP-1 (ATCC TIB-202, RRID: CVCL_0006), a previously optimized protocol was followed [16]. AO-stained yeast cells resuspended in RPMI with L-glutamine and phenol red (Nacalai Tesque, Japan) were added to PMA-differentiated THP-1 cells (3 × 10^5^ cells/well in a 12-well plate). Plates were then incubated for 2 h at 37 °C and 5% CO_2_. Cells were washed twice with cold PBS, detached using 0.25% trypsin-EDTA (Gibco, USA), and incubated for 5 min at 37 °C and 5% CO_2_ with occasional mixing. Fresh RPMI was added, and cells were washed twice with PEB buffer (1x PBS, 2.5 mM EDTA, 0.1% BSA), and resuspended in PEB buffer with 0.1% trypan blue. Because cells tend to clump, a cell strainer with a 40 μm mesh size (Funakoshi, Japan) was used before analysis. EC800 Flow Cytometry Analyzer with 488 nm and 561 nm lasers in combination with FL1 (525/50) and FL2 (585/40) bandpass filters were used to analyze at least 10,000 macrophages from each sample. The gating strategy was kept constant in all analyses. The early stage of the phagocytic event was analyzed from counted events in the green channel (FL1) whereas the late stage of the phagocytic event, wherein yeast cells are within acidified phagolysosomes, was counted from the red channel (FL2). Macrophages without added yeast serve as the control.

To induce lysis of macrophages, 1% Triton X-100 was added to macrophage cultures until a final concentration of 0.5%. Plates were kept at RT for 15 min with occasional mixing. Serial dilutions were prepared, and samples were spread-plated in YPD agar. After 48 h of incubation at 37 °C, the number of viable yeast cells was determined by CFU counting. The percentage of yeast cells killed by macrophages was calculated following the equation: % yeast killing = [(CFU of control well − CFU of test well) / CFU of control well x 100]. Wells containing only yeast cells serve as a control.

### Phagocytosis assay using RAW 264.7

This protocol is adapted from a previous study [17] but with slight modifications. RAW 264.7 (ATCC TIB-71, RRID: CVCL_0493) macrophages were seeded (100 µL of 2 × 10^6^ cells/mL) in a black-walled, clear-bottom 96-well plate (Greiner Bio-One, USA) in high glucose DMEM (Fujifilm Wako Pure Chemical Corporation, Japan) with 10% FBS and 1% penicillin-streptomycin. Media was removed after 2 h and 100 µL of the stained yeast suspension was added. Phagocytosis was allowed to proceed at 37 °C in 5% CO_2_ for 1 h. Then, 0.1% trypan blue was added to quench the fluorescence of yeasts that were not internalized. The number of internalized yeasts was measured by measuring fluorescence at 520 nm in a multi-well plate reader (Thermo Scientific Varioskan Lux). Using a 488 nm laser, the relative fluorescence unit (RFU) per well was measured and analyzed using the SkanIt v4.1 research edition software.

### Quantitative determination of cytokine concentration by ELISA

*Candida* and PMA-differentiated THP-1 macrophages were co-incubated for 24 h at 37 °C and 5% CO_2_. Cell culture supernatant was collected and kept at -80 °C before analysis. The concentration of cytokines (CCL3/MIP-1α, TNF-α, IL-10) was determined by sandwich ELISA following the manufacturer’s protocol (DuoSet ELISA, R&D Systems). All standards were analyzed in duplicates. Cytokine concentrations in the control and experimental samples were computed following the equation of the line generated in the standard curve. The final concentration was computed by deducting the cytokine concentration of the negative control (THP-1 only).

### Infection assay using silkworm

Silkworm infection experiments were performed as described previously [18] but with slight modifications. Third instar larvae of *Bombyx mori* (Fuyo x Tsukubane), purchased from Ehime Sanshu (Ehime, Japan), were fed with the antibiotic containing SilkMate 2 S (Nihon Nosan Kogyo Co. Ltd., Japan) until they developed to fifth instar larvae. Silkworms were bred in an incubator (EyelatronFLI-301 N, Rikakikai Co., Ltd, Japan) set at 27 °C. Silkworms were then fed with an antibiotic-free feed (10% mulberry leaf powder, 10% soybean flour, 2.5% agar) [19] for one day and starved a day before intrahemolymph injection. Yeast suspension (100 µL) containing 5 × 10^6^ yeast cells were injected into each larva using a 1 mL tuberculin syringe and a 27G × 3/4″ needle (Terumo Medical Corporation, Japan). The same volume of saline was injected as the control. Injected larvae were not fed and kept in an incubator set at 27 °C. Mortality was checked every 24 h for three days.

### Statistical analysis

For the β-glucan and mannan staining and phagocytosis assay, data were analyzed using GraphPad Prism version 9.4.0 for Windows (GraphPad Software, San Diego, California, USA). Means and standard deviations from at least three independent biological replicates were shown and the data were analyzed using a t-Test or one-way ANOVA with either Dunnett’s or Tukey’s multiple comparisons test. A *P* value of at least 0.05 is considered significant (*P < 0.05, **P < 0.01, ***P < 0.001, ****P < 0.0001).

For the infection assay, the experiment was independently performed thrice. In each replicate, 15 larvae were injected with *C. auris* grown in each condition while 10 larvae were injected with each of the control yeast strains. Survival data were evaluated by Kaplan-Meier analysis and statistical significance was calculated using a log-rank test. Calculated *P* values were adjusted following the Benjamini-Hochberg method using the “survminer” [20] package in R [21].

## Results

### Effect of environmental stimuli on β-glucan exposure levels in *C. auris*

**Fig. 1:**
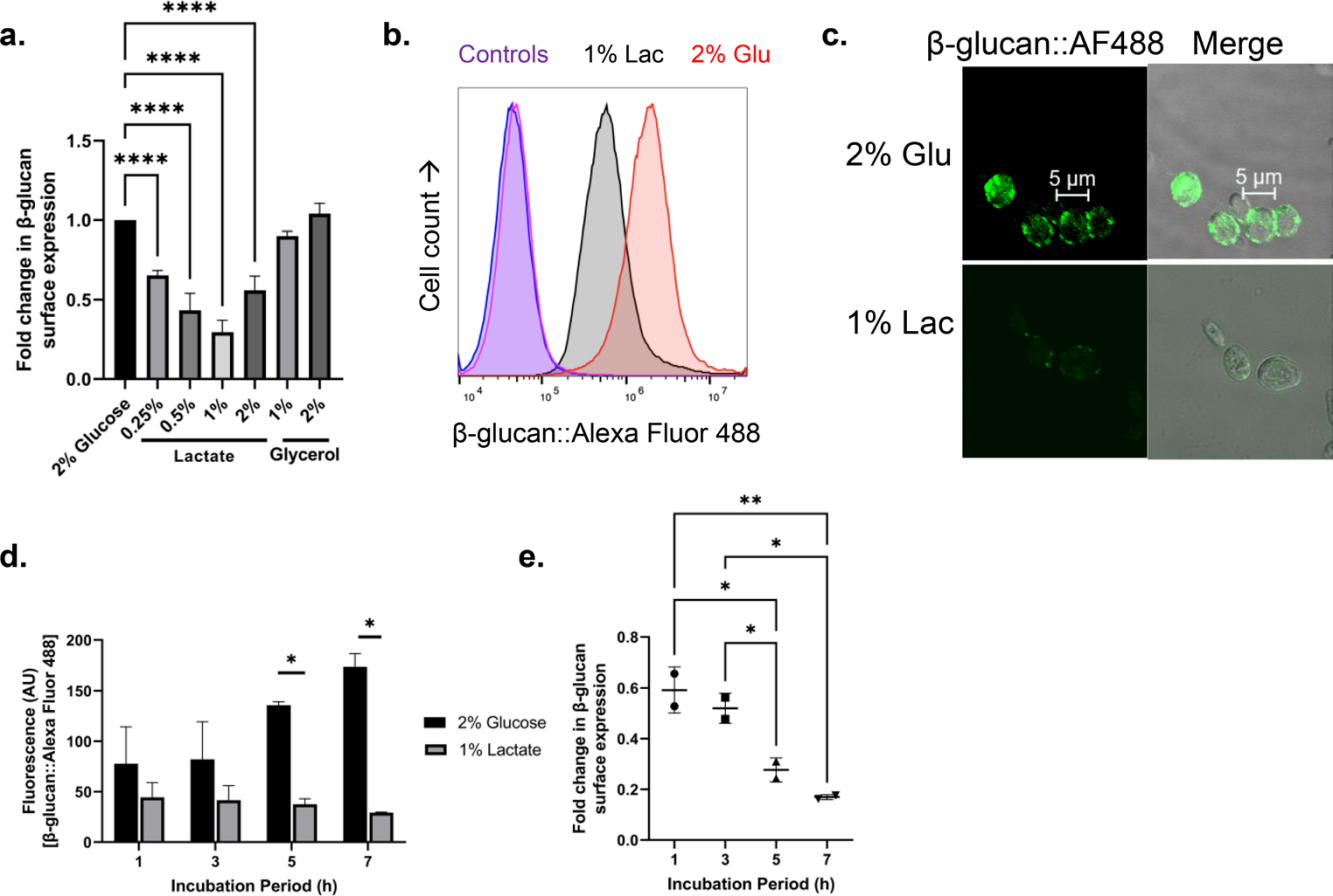
Lactate triggers reduction of exposed β-glucan in *C. auris.* (**A**) Effect of alternative carbon source on the expression of β-glucan on the surface of *C. auris* UI001 after a 5-h incubation at 37ºC. (**B**) Flow cytometric analysis of *C. auris* stained with Fc-hDectin-1a and Alexa Fluor 488-conjugated anti-Fc fluorochrome. Histogram shown is a representative of eight independent experiments showing consistent results. (**C**) Micrographs, which represent one out of two independent trials, showing *C. auris* β-glucan in green. (**D** and **E**) β-glucan exposures of *C. auris* from 1 h to 7 h of incubation. Bar graphs show the mean and SD from at least three independent biological replicates and analyzed through t-test (D) or one-way ANOVA with Dunnett’s (A) or Tukey’s (E) multiple comparisons test. * *P* < 0.05, ** P < 0.01, **** *P* < 0.0001

The effect of alternative carbon sources, such as lactate and glycerol, in the expression of β-glucan on the surface of *C. auris* cells was determined. Lactate and glycerol are abundant in the colon [22] and are significant physiological metabolites. Figure 1A-C show that in *C. auris* UI001, β-glucan amount on the surface of cells decreased in the presence of 0.25%, 0.5%, 1%, and 2% lactate (average fold change = 0.65, 0.43, 0.32, and 0.56, respectively). This shows that the decrease in β-glucan is triggered by lactate addition. To investigate β-glucan alteration at different times after the addition of lactate, fungal cells grown from 1 h to 7 h were isolated for the analysis of β-glucan. Figure 1D shows that for *C. auris*, the β-glucan::Alexa Fluor 488 fluorescence of cells in 1% lactate medium did not increase but this value in 2% glucose medium increased. This suggested that β-glucan production stopped in the presence of lactate. Furthermore, the proportion of β-glucan on the cell surface reduced gradually (Fig. 1E).

**Fig. 2:**
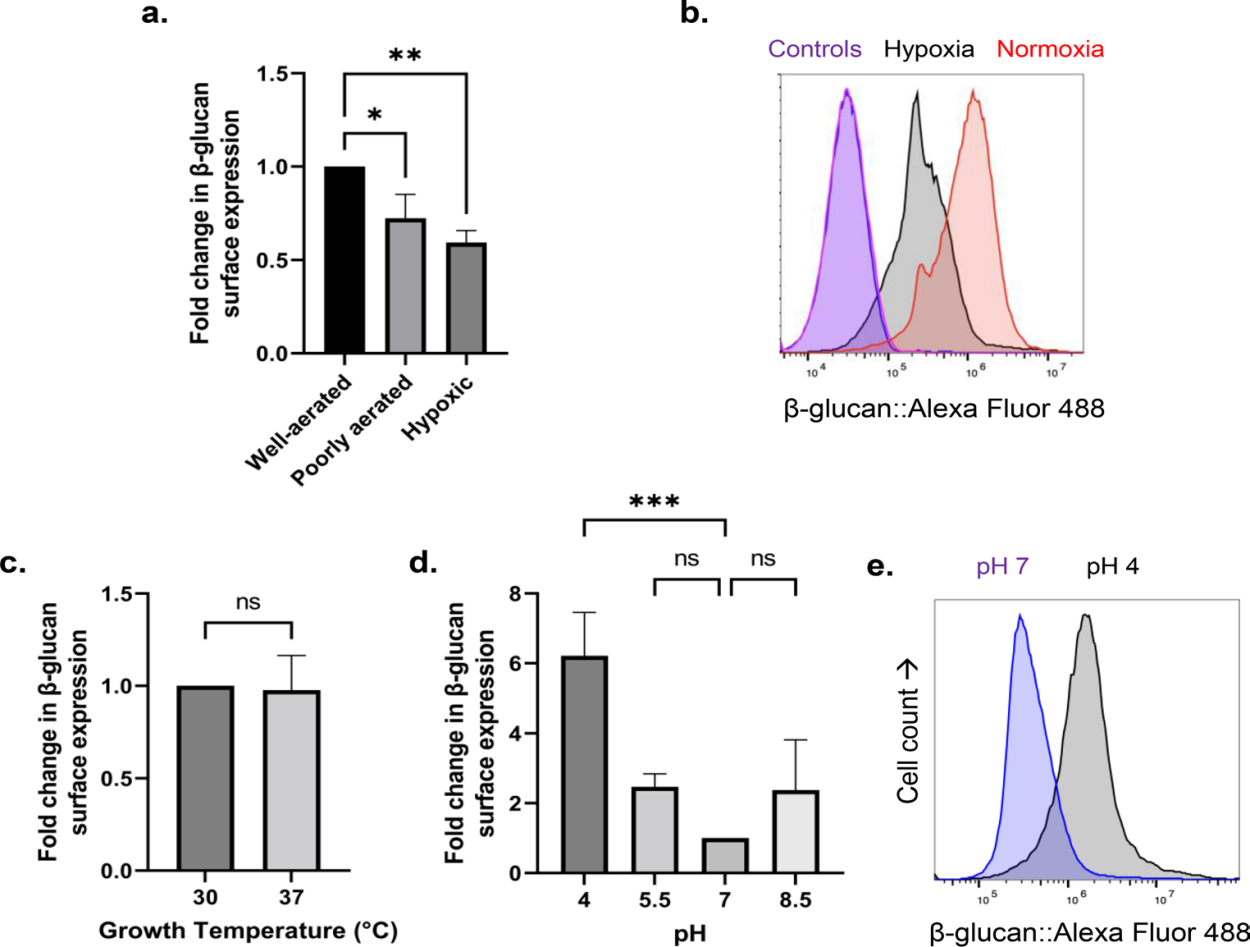
Effect of hypoxia, temperature, and pH on β-glucan surface exposure levels in *C. auris.* (**A, B**) Effect of environmental oxygen level, (**C**) ambient temperature and (**D**, **E**) pH on the surface expression of β-glucan in *C. auris* UI001 after a 5-h incubation at 37ºC. B and E shows the flow cytometric analysis of *C. auris* UI001 stained with Fc-hDectin-1a and Alexa Fluor 488-conjugated anti-Fc fluorochrome. Histogram shown is a representative of at least three independent experiments showing consistent results. Bar graphs show the mean and SD from three independent biological replicates analyzed through unpaired t-test (**C**) or one-way ANOVA with Dunnett’s multiple comparisons test (**A**, **D**). *P < 0.05, **P < 0.01, *** *P* < 0.001, ns = not significant

The effect of oxygen level on the β-glucan amount was determined next. As shown in Fig. 2A-B, the oxygen level during cell growth affects β-glucan production in *C. auris*, where there is a 41% reduction of the β-glucan during hypoxic growth. This phenomenon was previously observed in *C. albicans* [13].

In addition, the effect of culture temperature and pH on exposure levels of β-glucan were determined. *C. auris* showed the same exposure levels of β-glucan in both 30 °C and 37 °C and exhibited a similar β-glucan exposure profile to *C. albicans* [23, 24] when grown in different pH conditions (Fig. 2D-E). Cells grown at pH 4 display increased β-glucan levels compared to pH 7.0 (Fig. 2D).

**Fig. 3:**
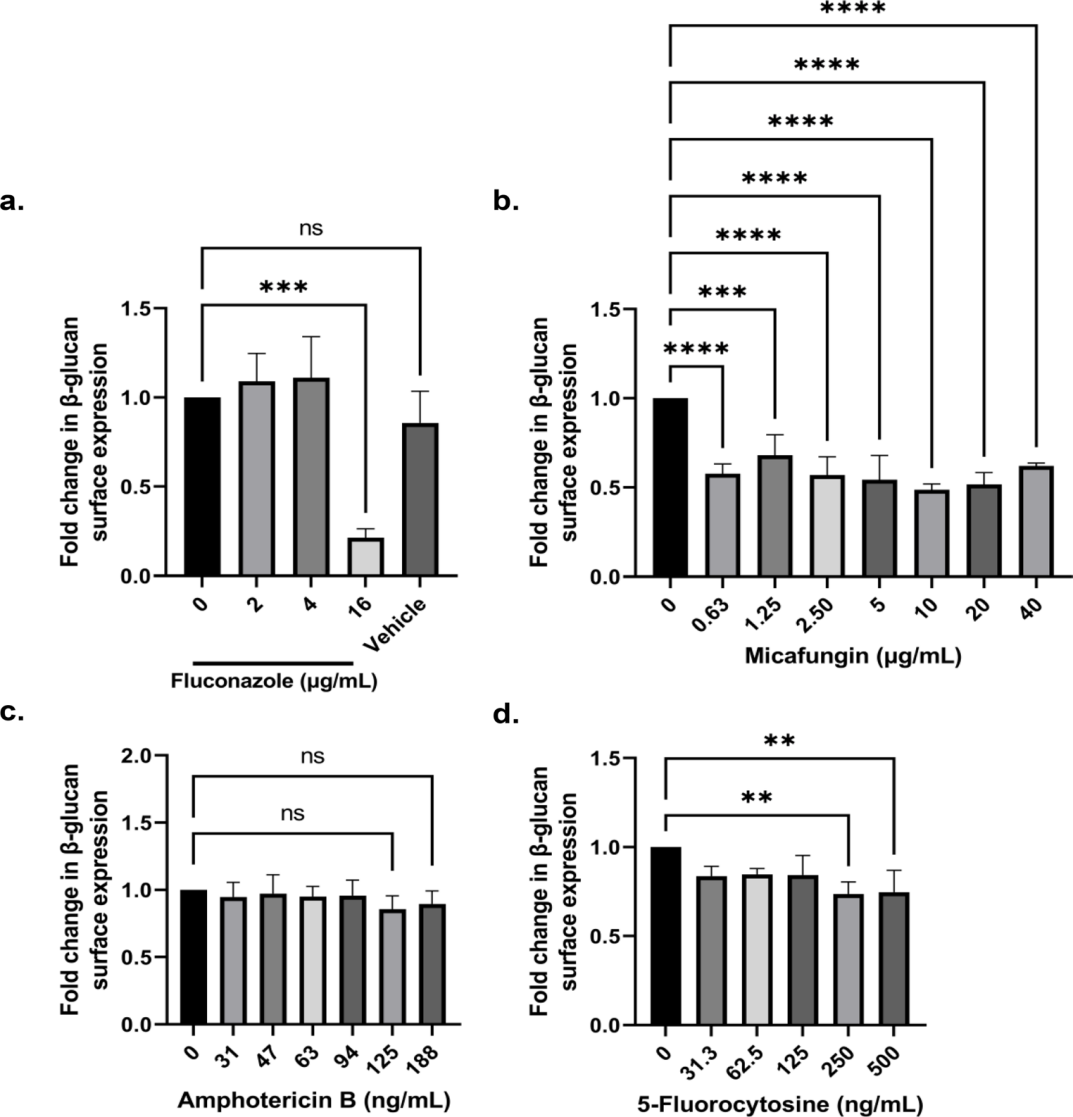
Sublethal concentrations of antifungals trigger reduction of β-glucan amount in *C. auris.* Effect of (**A**) Flc, (**B**) Mfg, (**C**) AmB and (**D**) 5FC on the surface exposure of β-glucan in *C. auris* UI001 after a 5-h incubation at 37ºC. Flc and AmB were resuspended in DMSO (“vehicle”) while Mfg and 5FC in sterile water. Bar graphs show the mean and SD from three independent biological replicates analyzed through one-way ANOVA with Dunnett’s multiple comparisons test. ** P < 0.01, *** *P* < 0.001, **** *P* < 0.0001, ns = not significant

Exposure of *Candida* to antifungal drugs has been previously shown to affect the expression of proteins related to the cell wall [25]. Therefore, to clarify the influence of antifungals on the exposure levels of β-glucan in *Candida* species, the amount of β-glucan on the surface of *C. auris* exposed to sublethal concentrations of antifungals (azole, echinocandin, polyene, and pyrimidine analog) was determined. As shown in Fig. 3, the decrease in β-glucan exposure was observed in the presence of 16 µg/mL fluconazole (79% reduction) and ≥ 0.63 µg/mL of micafungin (maximum 42% reduction). There is also a slight reduction of β-glucan in the presence of 250–500 ng/mL 5-fluorocytosine but no change in the presence of amphotericin B. *C. albicans* was reported to have increased β-glucan exposure under the presence of fluconazole and amphotericin B [12]. The observations in this *C. auris* strain are contradicting because β-glucan reduction occurred in the presence of fluconazole and micafungin, and amphotericin B did not influence the amount of β-glucan.

The β-glucan alteration phenomenon of other *C. auris* strains belonging to a different clade was investigated. Unlike UI001 (Clade I), lactate-induced β-glucan is not observed in *C. auris* ATCC MYA-5001 and MYA-5002, a representative of Clades II and III, respectively (Fig. S1A). The low oxygen condition could trigger reduced β-glucan in MYA-5001 but not in MYA-5002 (Fig. S1B). In addition, the change of β-glucan amount in different pH conditions did not show a similar result as the UI001 strain (Fig. S1C). From these results, the β-glucan exposure profiles of *C. auris* may be different among clades.

### Relation between mannan and β-glucan exposures on the surface of *C. auris* cells

**Fig. 4:**
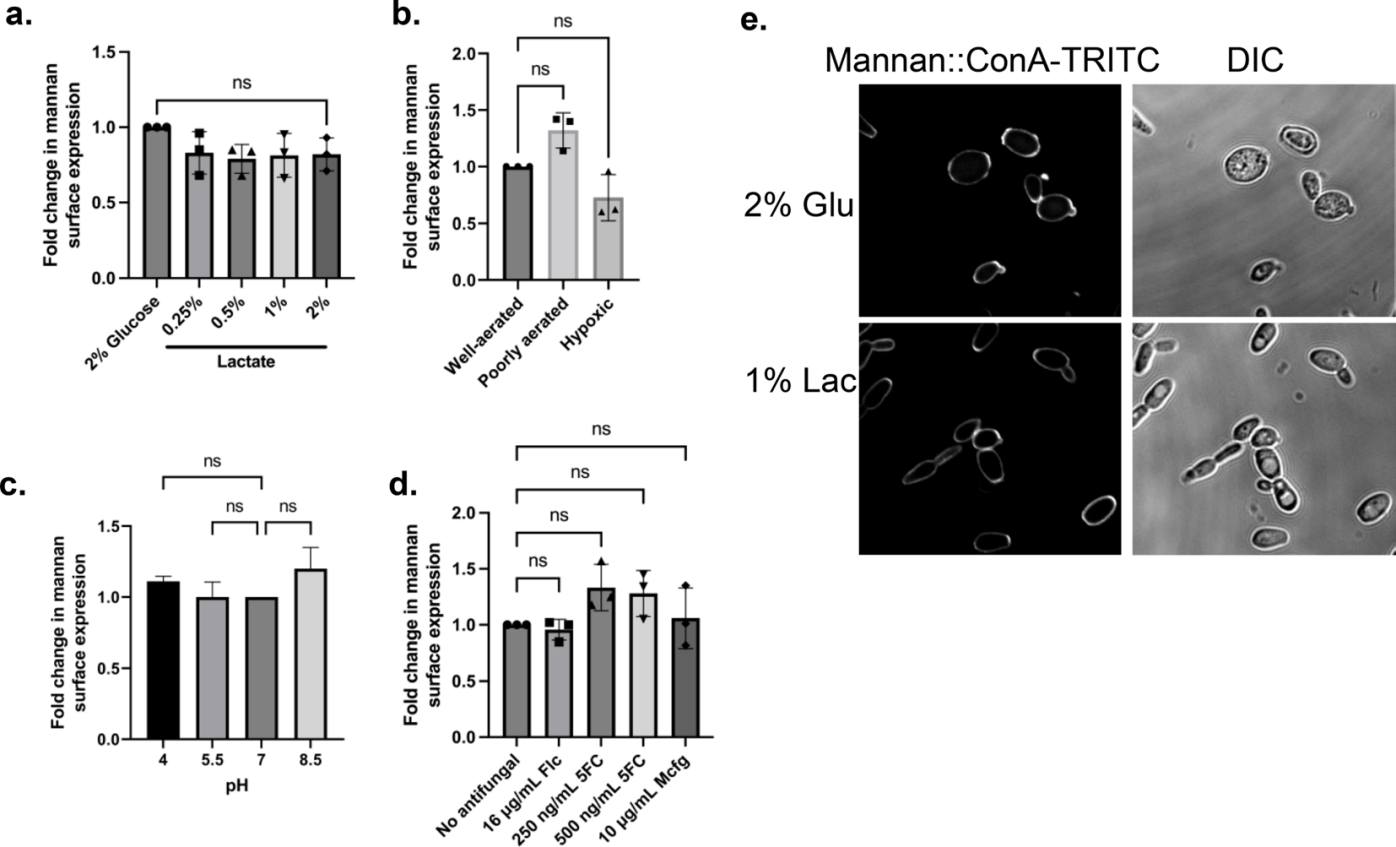
Mannan exposure profiles of *C. auris* in varying physiologically relevant environmental conditions. Mannan surface exposure levels of *C. auris* UI001 due to **A**) lactate, (**B**) low oxygen, (**C**) pH, and (**D**) antifungals after a 5-h incubation at 37ºC, and (**E**) micrograph showing *C. auris* mannan. Bar graphs show the mean and SD from three independent biological replicates analyzed through one-way ANOVA with Dunnett’s multiple comparisons test. ns = not significant

To investigate whether β-glucan alteration observed in *C. auris* is related to mannan surface exposure, mannan exposure profiles were analyzed by subjecting cells to the same culture conditions and analyzing mannan::TRITC fluorescence by flow cytometry. Lactate, oxygen concentration, pH, and antifungals did not affect mannan exposure in *C. auris* UI001 (Fig. 4A-D). *C. auris* was stained and viewed on a confocal microscope (Fig. 4E). It is clear from the microscopy images that there is no change in mannan levels after a 5-h incubation in a medium containing lactate.

The two type strains of *C. auris* also exhibit a similar result as the UI001 strain, although the mannan amount of MYA-5001 is increased in both acidic and alkaline conditions (Fig. S2).

Overall, the data presented reveal that lactate- and hypoxia-induced reduction of exposure levels of β-glucan observed in *C. auris* are not due to thickening of mannan and that exposure levels of β-glucan and mannan do not follow an inverse pattern.

### Effect of β-glucan change in the surface of *C. auris* on the host immune response

**Fig. 5:**
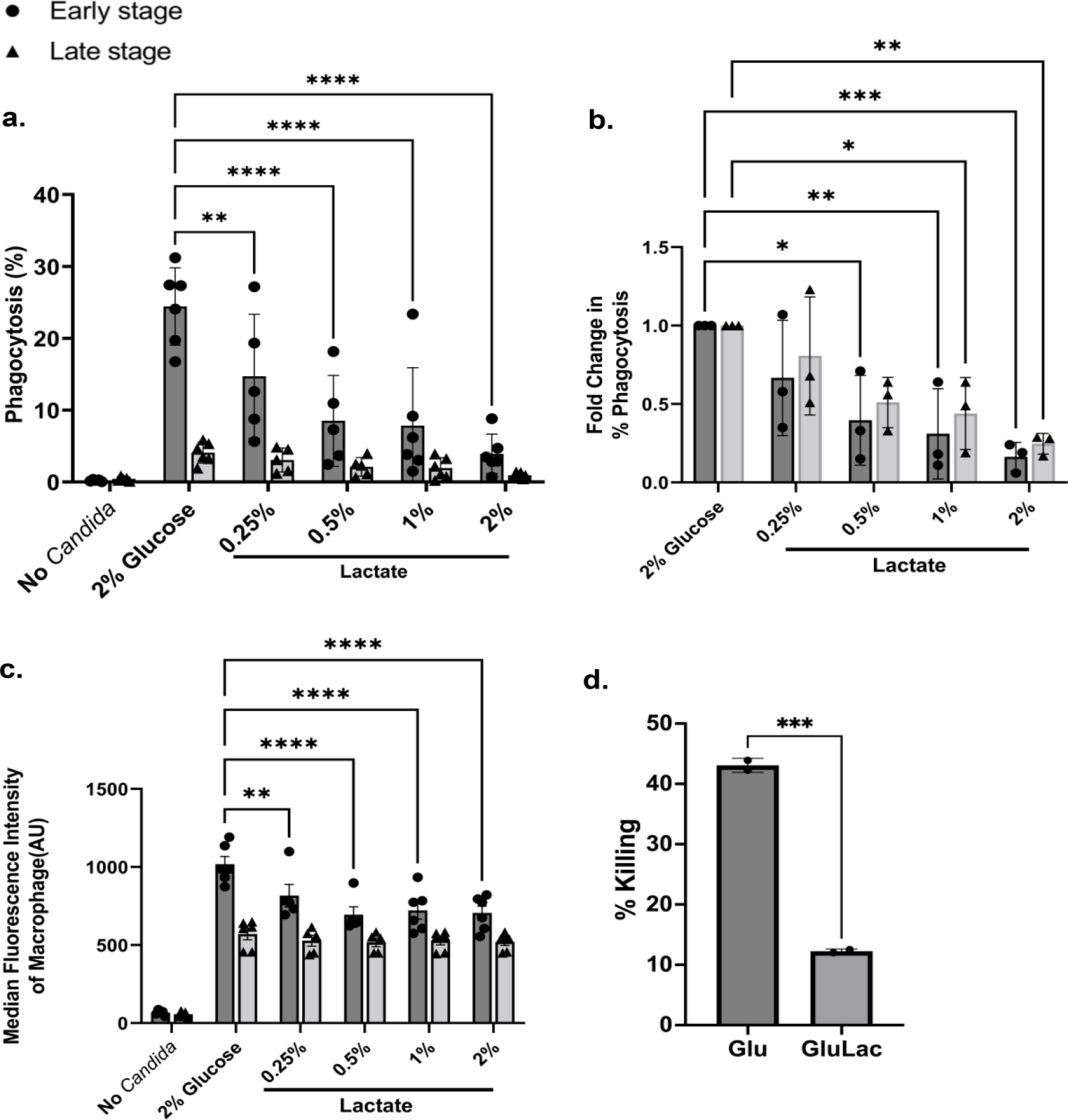
Lactate-induced reduction in β-glucan amount triggers immune evasion from THP-1 macrophages. *C. auris* UI001, pre-cultivated in glucose only or with varying amounts of lactate, was co-incubated with PMA-differentiated THP-1 for 2 h. (**A**) Efficiency of phagocytosis was measured by flow cytometric analysis of macrophages. (**B**) The fold change in phagocytosis is the ratio of % phagocytosing macrophages versus the control (2% Glucose). (**C**) Median fluorescence intensity of phagocytosing macrophages signifying the quantity of phagocytosed fungal cells. (**D**) The % of *C. auris* killed by macrophages after 2 h where GluLac = 1% Glu+1% Lac. Early-stage (solid circle) and late-stage phagocytosis (solid triangle) was measured using the FL1 and FL2 bandpass filters, respectively. Bar graphs show the mean and SD from three independent biological replicates analyzed through two-way ANOVA with Dunnett’s multiple comparisons test (**A**, **B**, **C**) or unpaired t-test (**D**). * *P* < 0.05, ** P < 0.01 *** P < 0.001, **** P < 0.0001

The β-glucan alteration of *Candida* species is thought to be one of the fungal activities to evade recognition from the host immune cells. We analyzed the phagocyte activity of mammalian macrophages against *C. auris* to determine the effect of β-glucan exposure on the response of these macrophages. Figure 5A shows that 24% of the macrophages phagocytosed at least one cell of glucose-grown *C. auris* whereas only 4% for the lactate-grown *C. auris*. Figure 5B shows that lactate-induced β-glucan decrease in *C. auris* triggered up to 84% and 75% reduction in early-stage and late-stage phagocytosis, respectively, while Fig. 5C shows that macrophages take in more glucose-grown fungal cells than lactate-grown cells. Pre-cultivation in increasing amounts of lactate led to more reduction in phagocytosis.

Since *C. glabrata* was shown to be able to survive and replicate inside macrophages, eventually leading to macrophage lysis due to fungal load [26], the ability of macrophages to kill *C. auris* cells was determined. Figure 5D shows that macrophages can kill more than 40% of fungal cells grown in a glucose medium but only 10% of the lactate-grown cells. This demonstrates that the reduction of β-glucan in the cell wall of *C. auris* led to a reduction of killing inside the macrophages.

**Fig. 6:**
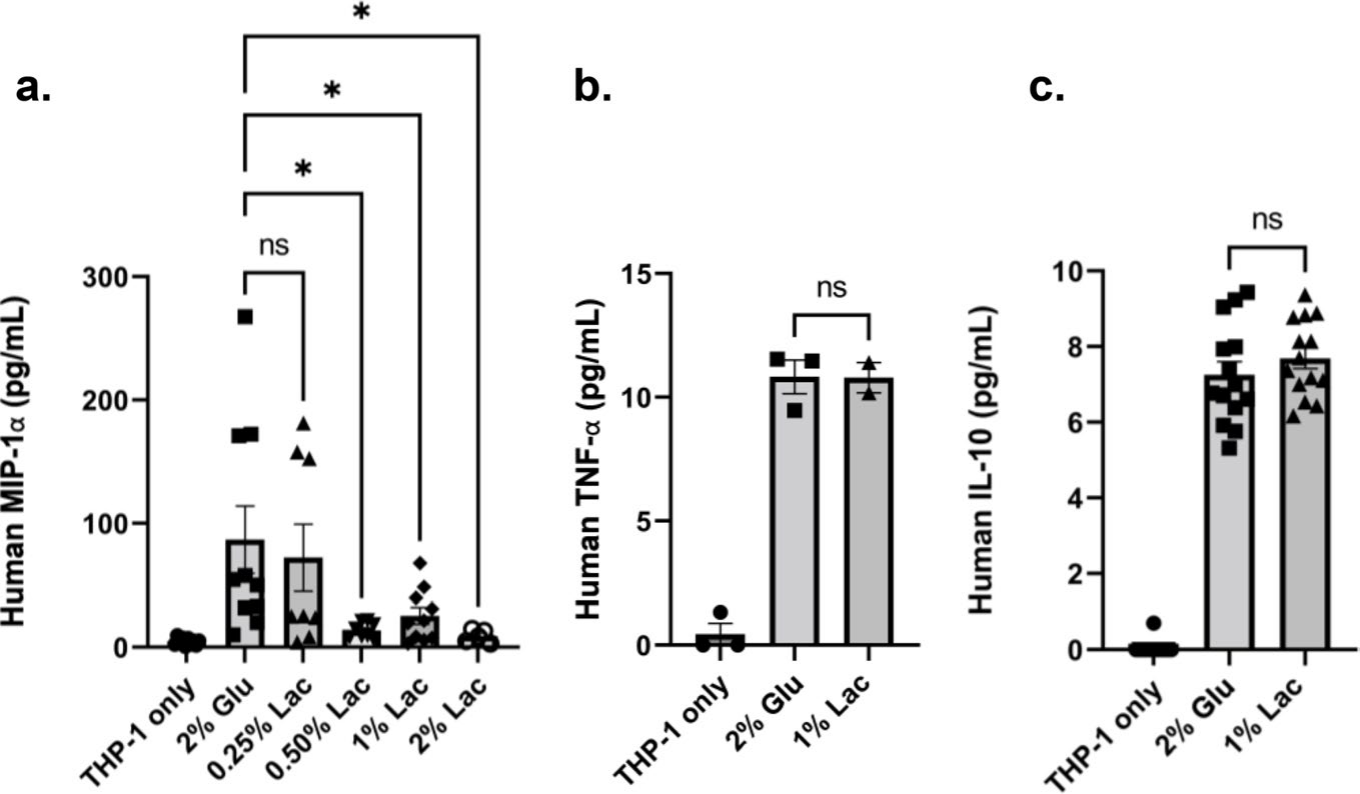
Lactate-induced reduction of β-glucan amount in *C. auris* triggers reduced CCL3/MIP-1α production but not TNF-α and IL-10 in phagocytosing THP-1 macrophages. ELISA-based quantification of (**A**) CCL3/MIP-1α, (**B**) TNF-α, and (**C**) IL-10 produced by PMA-differentiated THP-1 macrophages after co-incubation with *C. auris* previously grown in glucose only or with varying amounts of lactate. Bar graphs show the mean and SD from at least three independent biological replicates analyzed through one-way ANOVA with Dunnett’s multiple comparisons test. * *P* < 0.05, ns =not significant

The effect of changes in the cell wall architecture of *C. auris* on the immune factor production of macrophages was also investigated. Figure 6 shows that, compared with *C. auris* cultivated in glucose medium, *C.auris* UI001 grown with ≥ 0.5% lactate, where cells exhibit a 57% reduction in β-glucan, led to an 84% reduction in CCL3/MIP-1⍺ production, but no significant effect on TNF-⍺ and IL-10 production.

**Fig. 7:**
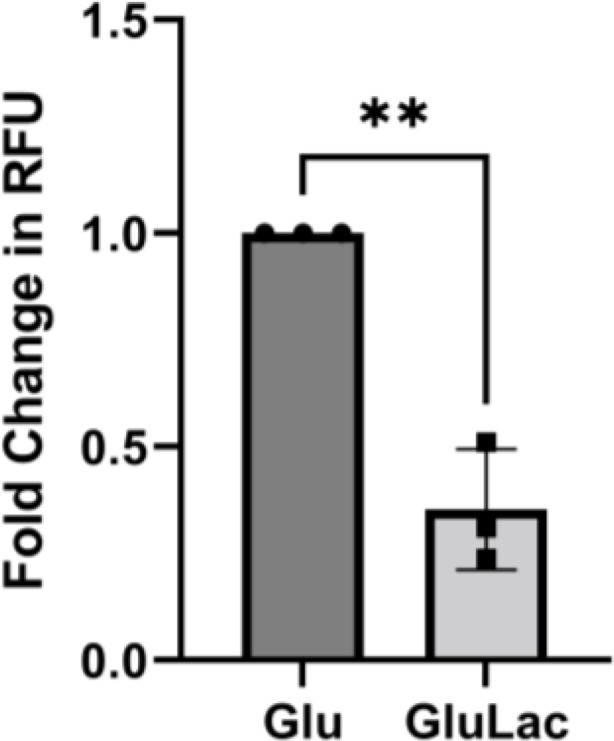
Lactate-induced reduction in β-glucan amount triggers immune evasion from RAW 264.7 macrophages. *C. auris* UI001 cells pre-cultivated in glucose only (Glu) or 1% glucose-1% lactate (GluLac) were co-incubated with RAW 264.7 macrophages, at a macrophage-to-yeast ratio of 1:6. for 1 h. Relative fluorescence units (RFU) were measured in a multi-well plate reader. The fold change in phagocytosis, measured by computing the fold change in RFU, is the ratio of % phagocytosing macrophages infected with GluLac-grown fungal cells versus the control. Bar graph shows the mean and SD from three independent biological replicates analyzed through unpaired t-test. ** P < 0.01

The effect of β-glucan reduction phenomenon on the phagocytic capacity of murine macrophage cell line RAW 264.7 was also experimented with to ensure that the observed reduction in phagocytosis is exhibited regardless of the type of macrophage. Figure 7 shows that murine macrophages were unable to efficiently phagocytose lactate-grown *C auris*. There is a 65% reduction in the phagocytosis of *C. auris* pre-cultivated in a lactate-containing media. This result was similar to that of PMA-differentiated THP-1 macrophages (Fig. 5).

### Effect of β-glucan alteration in *C. auris* on its virulence on domestic silkworm

To determine if the decrease in β-glucan influences the virulence of *C. auris*, we injected *C. auris* into silkworm larvae. The experiment showed that there is a difference in the survival of larvae injected with *C. auris* pre-cultured in glucose only vs. the same strain but pre-cultured in glucose-lactate (*P* = 0.042), where median survival is 48 h and 24 h, respectively. From the result, it was suggested that β-glucan reduction in *C. auris* enhanced its virulence in silkworm larvae. Moreover, after the 72-hour observation period, all uninjected silkworm larvae, as well as those injected with 0.6% saline, survived.

**Fig. 8:**
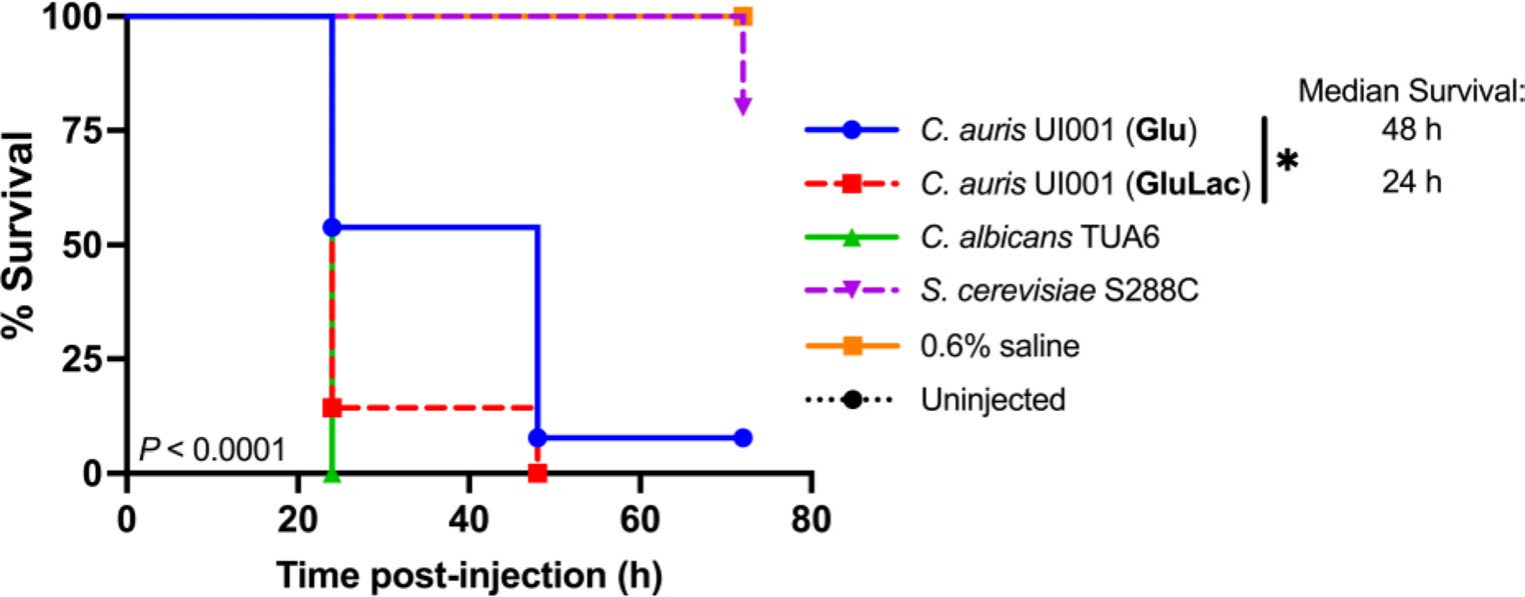
Reduction of β-glucan amount in *C. auris* leads to increased virulence in silkworm infection model. Kaplan-Meier survival curve for silkworm larvae following infection with 5 × 10^6^ cells of indicated strains. The graph represents data from three independent biological replicates. In each trial, 15 larvae were injected with *C. auris* precultured in each condition, 5 larvae were injected with each of the control yeast strains and 5 larvae for each of the experimental controls. Statistical significance was calculated using a log-rank test followed by BH correction of *P* values: * *P* < 0.05. Glu= 2% glucose; GluLac =1% glucose plus 1% lactate

## Discussion

*Candida* species are commensal organisms that are naturally part of the human microbiome. However, several factors could trigger some cells to invade mucosal surfaces and cause candidiasis [2]. Indeed, some *Candida* species develop effective immune evasion strategies to permit colonization of an immunologically competent host.

Interestingly, this study showed that an alternative carbon source like lactate, as well as low oxygen environment and sublethal concentrations of antifungals, trigger reduction of exposure levels of β-glucan in *C. auris* belonging to Clade I. Previous reports already revealed that carbon sources from the host cells affect host-pathogen interactions in *C. albicans* [27] but to date, this is the first study that reported lactate-induced decrease in β-glucan in *C. auris*.

The pathway controlling lactate-induced β-glucan masking in *C. auris* has not been fully elucidated. However, generally, β-glucan masking pathways differ between *C. auris* and *C. albicans* [28, 29]. Genes coding for proteins involved in β-glucan masking in *C. albicans* [14, 30, 31] have orthologs in *C. auris* based on the *Candida* Genome database (http://www.candidagenome.org). *C. auris* has orthologs of the following genes in *C. albicans*: *GPR1* (B9J08_002066), *CRZ1* (B9J08_002096), *ENG1* (B9J08_002109), and *XOG1* (B9J08_003251). The systematic name of the gene in *C. auris* is indicated in parenthesis.

The effect of oxygen level on β-glucan production on the surface of *Candida* sp. was first described by Pradhan et al. [13]. Hypoxia is an important condition observed in the lower gastrointestinal tract and inflamed tissues [32]. Previous studies showed that several pathogenic *Candida* species show varying adaptations to hypoxic environments and that most strains of *C. albicans, C. krusei*, and *C. tropicalis* showed masking of β-glucan whereas *C. auris*, as well as *C. parapsilosis* and *C. glabrata*, showed variable results. In contrast, most strains of *C. guilliermondii* do not exhibit β-glucan masking [13].

Earlier studies determined how *Candida* cell wall architecture is influenced by environmental pH [12, 23, 24]. To mimic the pH conditions in the gastrointestinal tract, fungal cells in this study were grown in YPD with the following pH: 4, 5.5, 7, and 8.5. These pHs represent the upper and lower stomach which is around pH 4, the small intestine which is about pH 7.0, and the colon which is nearly pH 8.5 [33]. Furthermore, relatively low ambient pH is associated with vulvovaginal niches, where certain *Candida* species are known to thrive. Contrary to our observation, a clinical strain of *C. auris* (JCH15448-1) did not show any pH- and time-dependent β-glucan unmasking [24]. We recommend checking for chitin exposure levels across a wide range of pH, as unmasking of cell wall in *C. albicans* may induce non-protective hyperactivation of the immune system during growth in acidic environment [23].

For the effect of antifungals, this study is the first to report the reduction of β-glucan due to fluconazole. However, a concentration of 16 µg/mL fluconazole, which is shown to trigger a reduction in β-glucan, might not be present in actual patients of candidiasis. Fluconazole levels in plasma and human tissue and body fluids are approximately below this level [34]. For the effect of echinocandins on *C. auris* β-glucan, our observation is supported by recent findings of a Clade 1 *C. auris* isolate (B11219) exhibiting reduced surface β-glucan following micafungin treatment [28].

The growth of *C. auris* strains used in this study is the same as the control under all environmental conditions tested except during low oxygen. MFI values of samples grown in each condition is summarized in a supplemental table (Table S1). Overall, *C. auris* strain used in this study (UI001) exhibits a similar β-glucan expression profile to *C. albicans* SC5314 [12–14, 23, 24]. *C. auris* belonging to Clade I exhibits a highly similar cell wall composition and organization to *C. albicans* SC5314 [16] which could partly explain the similar environment-triggered β-glucan exposures of these strains.

In line with previous reports, alteration of β-glucan levels occurs due to cell wall remodeling. To date, little to no studies regarding the dynamics between β-glucan and mannan exposures have been performed on *C. auris*. Mannan is found on the outermost part of the cell and could possibly affect exposure levels of β-glucan [35], thus affecting the recognition of fungal pathogens by the host immune system. It is interesting to find out if thickening of the mannan is the cause of the reduction in β-glucan in *C. auris*. Even more so when *C. auris*-induced late innate immune activation is revealed to be elicited primarily by structurally unique *C. auris* mannoproteins [36]. We have observed that thickening of mannan is not the cause of the reduction of β-glucan exposure in *C. auris*. Several studies have hinted at this [14, 31] and so, we hypothesize that although there is no direct correlation between β-glucan masking and mannan levels in *C. auris*, there is a possibility that the change of mannan structure may lead to less accessibility of β-glucan. To date, there are no in-depth studies that determined the cell surface expression of mannan in *C. auris*, specifically by concanavalin A-TRITC staining and flow cytometry analysis.

This study showed that different strains of *C. auris* have different cell wall responses to varying environmental conditions (Fig. S1 and Fig. S2). Other studies have also observed differences between *C. auris* strains [28, 36, 37]. We believe that mechanisms for environment-triggered cell wall remodelling may be different between strains regardless if they belong to the same clade. To date, there is no direct explanation about this phenomenon and thus serves as avenue for future research.

The effect of β-glucan alteration on the host immune response was determined. All *Candida* species display PAMPs on the cell surface but the immune reactivity of fungal cell surfaces is not correlated with the relatedness of fungal species [38]. This means that past findings on host-pathogen interaction studies mainly performed using *C. albicans*, may not be true for other species. So, current knowledge about *C. albicans* cell wall and its interaction with innate immune cells cannot be extrapolated to *C. auris* [16]. Indeed, an earlier study demonstrated that neutrophils preferentially target *C. albicans* in mixed cultures with *C. auris*, and *C. auris* evaded neutrophil capture via neutrophil extracellular trap formation [29, 39, 40]. Unlike neutrophils, macrophages can recognize and phagocytose *C. auris* [39] and are considered the main immune cell populations responsible for host defense against systemic candidiasis [11]. A parameter used to evaluate the host immune response is cytokine production upon infection since immunomodulation with cytokines can enhance the antifungal activity of immune cells and upregulate protective T-helper type 1 adaptive immune responses [41]. This study used human monocytic cell line THP-1 and murine macrophage cell line RAW 264.7. THP-1 monocytes differentiated into macrophages through the addition of PMA can mimic the response of human primary macrophages [42]. On the other hand, RAW 264.7 cells are a commonly used model of mouse macrophages for the study of cellular responses to microbes and their products.

In vitro infection assays were performed to determine the effect of β-glucan alteration on the rate of phagocytosis of *C. auris.* We focused on the effect of lactate-induced decrease in β-glucan since lactate (1%) triggers the most reduction in β-glucan among all conditions tested (Fig. 1A). To have a full picture of the effect of PAMP exposure levels on the immune response, chitin levels can also be determined since reduced cell wall chitin is shown to affect late-phase cytokine response in another species, *C. albicans* [43]. We have observed low production of cytokines by THP-1 macrophages infected with *C. auris* (Fig. 6). In a similar study, peripheral blood mononuclear cells fail to induce a potent pro-inflammatory cytokine response against *C. auris* [39]. Furthermore, another study showed that human M1-activated monocyte-derived macrophages stimulated less CCL3/MIP-1⍺ and less TNF-α after infection with lactate-grown *C. albicans* compared to glucose-only-grown cells [14]. In this study, macrophages exposed to *C. auris* cells exhibiting lactate-induced reduction in β-glucan produced less of CCL3/MIP-1⍺ but not TNF-α and IL-10. CCL3/MIP-1⍺ is a chemotactic cytokine produced by cells during infection or inflammation. Compared to TNF-α and IL-10, CCL3/MIP-1⍺ plays an important role in recruiting various cells such as monocytes, macrophages, lymphocytes, and eosinophils via the CCR1 or CCR5 receptor [44], thereby strengthening the immune response against a fungal infection.

Aside from an in vitro infection assay, an in vivo approach was conducted to determine the effect of lactate-induced β-glucan change in *C. auris* on a host. Due to some limitations in institutional facilities, an in vivo work on mice was not performed to observe the pathogenesis of *C. auris* and recruitment of immune cells toward the infection site. Several infection studies in which *Candida* sp. was injected into invertebrate hosts such as silkworm [18, 45, 46] have been carried out. Silkworms allow in vivo examinations of phagocytic cell function [46] and investigation of molecular mechanisms of infection by human fungal pathogens [18]. The larvae can thrive at mammalian physiologic temperature (37 °C), which allows for the expression of relevant temperature-regulated virulence factors [47]. Fungal cell wall components such as mannan and β-glucan can activate the immune system of *B. mori*. The β-1,3-glucan recognition protein (βGRP) on the surface of silkworm hemocyte binds to β-1,3-glucan leading to activation of the prophenoloxidase cascade resulting in the production of quinones and melanin, which then manifests as blackening of the injected larvae [48]. Figure 8 shows that lactate-induced change of β-glucan in *C. auris* leads to enhancement of virulence of cells, probably due to a reduction in recognition by hemocytes leading to less efficient clearing of the pathogen. Overgrowth of *C. auris* due to immune evasion may have caused the death of the larvae as the growth of fungi can largely contribute to the death of the silkworms. Our observation is in line with other studies where hypoxia-induced reduction in the β-glucan of *C. albicans* led to increased virulence in a *C. elegans* infection model [32]. Conversely, an increased level of PAMP exposure in low pH-grown *C. albicans* correlates with higher pathogenicity [24]. The infection experiment result complements the data presented in Figs. 5 and 7, where lactate-induced reduction in β-glucan in *C. auris* UI001 triggered a significant reduction in phagocytosis by THP-1 and RAW 264.7 macrophages. The phagocytic ability of these types of macrophages may not be the same as the immune cells of silkworms, but considering that silkworms can recognize β-1,3-glucan via βGRP found on the surface of silkworm hemocyte, we hypothesize that alteration of β-glucan lead to immune evasion, thus leading to increased virulence of *C. auris*.

## Conclusion

This study provides evidence that β-glucan alteration occurs in the emerging fungal pathogen, *C. auris*. The presence of lactate, lower oxygen levels, and sublethal concentration of fluconazole led to a decrease in β-glucan in *C. auris*. On the contrary, low pH triggers an increase in exposure levels of β-glucan. Our results reveal that mannan exposure levels are independent from β-glucan. This clearly supports the idea that the *Candida* cell wall is highly dynamic and diverse, and changes observed are strain specific.

The alteration of cell wall β-glucan in *C. auris* affected the immune response against the pathogen. Lactate-induced β-glucan alteration in *C. auris* triggered up to 84% (early stage) and up to 75% (late stage) reduction in phagocytosis by PMA-differentiated THP-1 macrophages and a 65% reduction in early-stage phagocytosis by RAW 264.7 macrophages. In addition, the killing of lactate-grown cells decreased four-fold compared to that of glucose-only grown cells.

Silkworms were used in the in vivo infection assays. Silkworm larvae appear to have a lower chance of surviving if *C. auris* cells exhibiting reduced β-glucan exposure were injected. This may be explained by the reduction in the recognition of yeast cells by βGRP on the surface of hemocytes leading to immune evasion and enhanced survival and overgrowth of *C. auris.* This observation supports the in vitro phagocytosis assay result using human and mouse macrophages. Therefore, lactate-induced reduction of β-glucan in *C. auris* potentially triggers immune evasion leading to increased virulence of *C. auris* in a host organism.

This study sheds light on the dynamics of β-glucan exposure in *C. auris* which could then help researchers comprehend the immune response against this emerging and critical priority pathogen. Ultimately, this research aims to help develop novel strategies to combat one of the most multidrug-resistant pathogens, *C. auris.*

## Electronic supplementary material

Below is the link to the electronic supplementary material.

Supplementary Material 1

## Data Availability

No datasets were generated or analysed during the current study.
