# Supplementary material for: Alteration of β-glucan in the emerging fungal pathogen Candida auris leads to immune evasion and increased virulence
Source: Med Microbiol Immunol. 2024 Jul 5;213(1):13. doi: 10.1007/s00430-024-00795-y (PMC11226559; doi:10.1007/s00430-024-00795-y)
Supplement: Supplementary file 1 — Supplementary Material 1 [file 430_2024_795_MOESM1_ESM.docx]

**MEDICAL MICROBIOLOGY AND IMMUNOLOGY**

**Alteration of β-glucan in the emerging fungal pathogen *Candida auris* leads to immune evasion and increased virulence**

Shiela Marie Gines Selisana^1^, Xinyue Chen^1^, Eny Mahfudhoh^1^, Anom Bowolaksono^2^, Anna Rozaliyani^3^, Kanami Orihara^1^, and Susumu Kajiwara^1*^

^1^School of Life Science and Technology, Tokyo Institute of Technology, Nagatsuta-cho, Midori-ku, Yokohama, Kanagawa 226-8501, Japan

^2^Faculty of Mathematics and Science, University of Indonesia, Depok, 16424, Indonesia

^3^Faculty of Medicine, University of Indonesia, Jakarta, 10430, Indonesia

^*^ [kajiwara.s.aa@m.titech.ac.jp](mailto:kajiwara.s.aa@m.titech.ac.jp) (corresponding author)

**MYA-5002 (Clade III)**

**MYA-5001 (Clade II)**

**a.**

**b.**

**c.**

***C. auris* ATCC:**


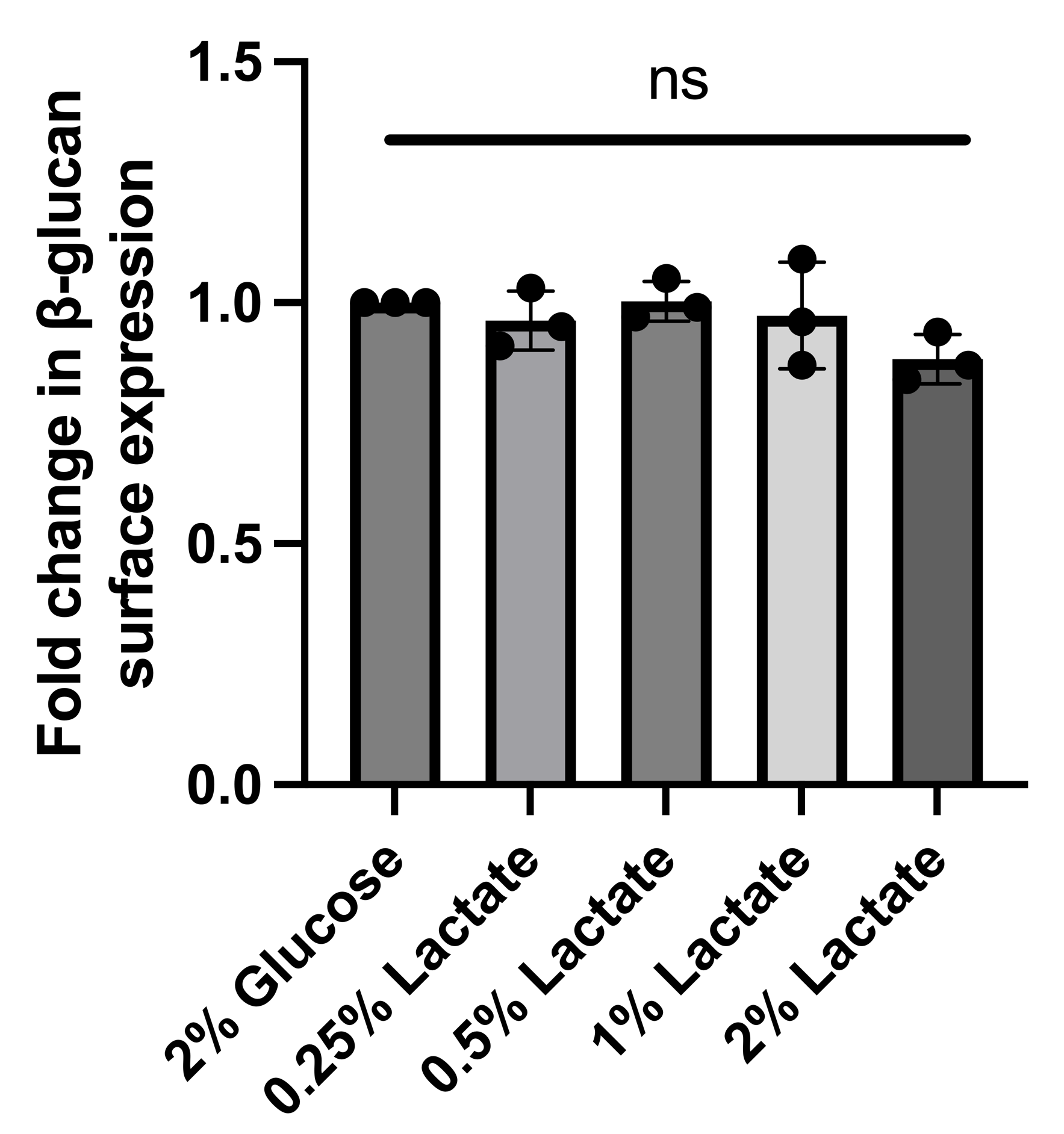

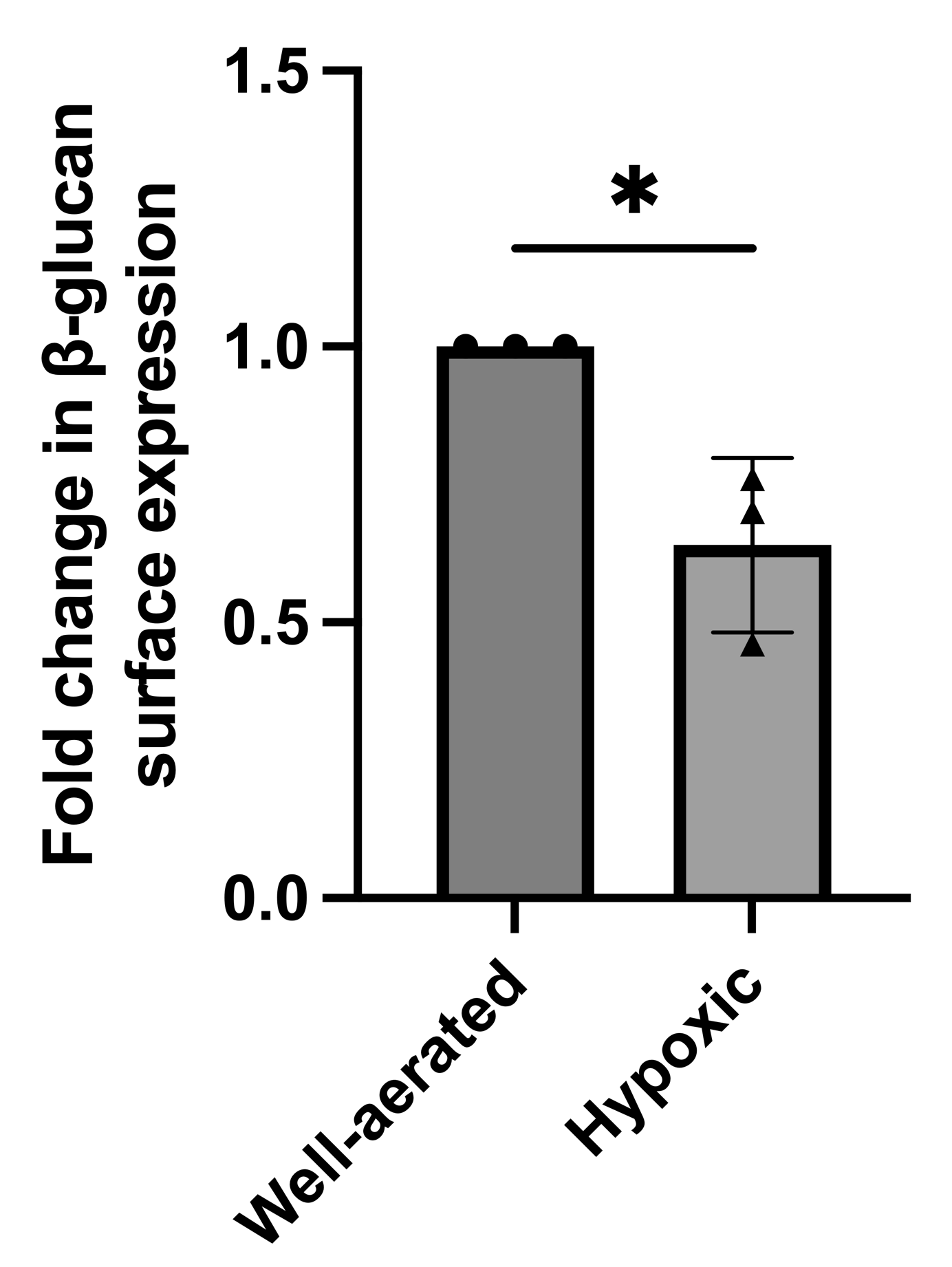

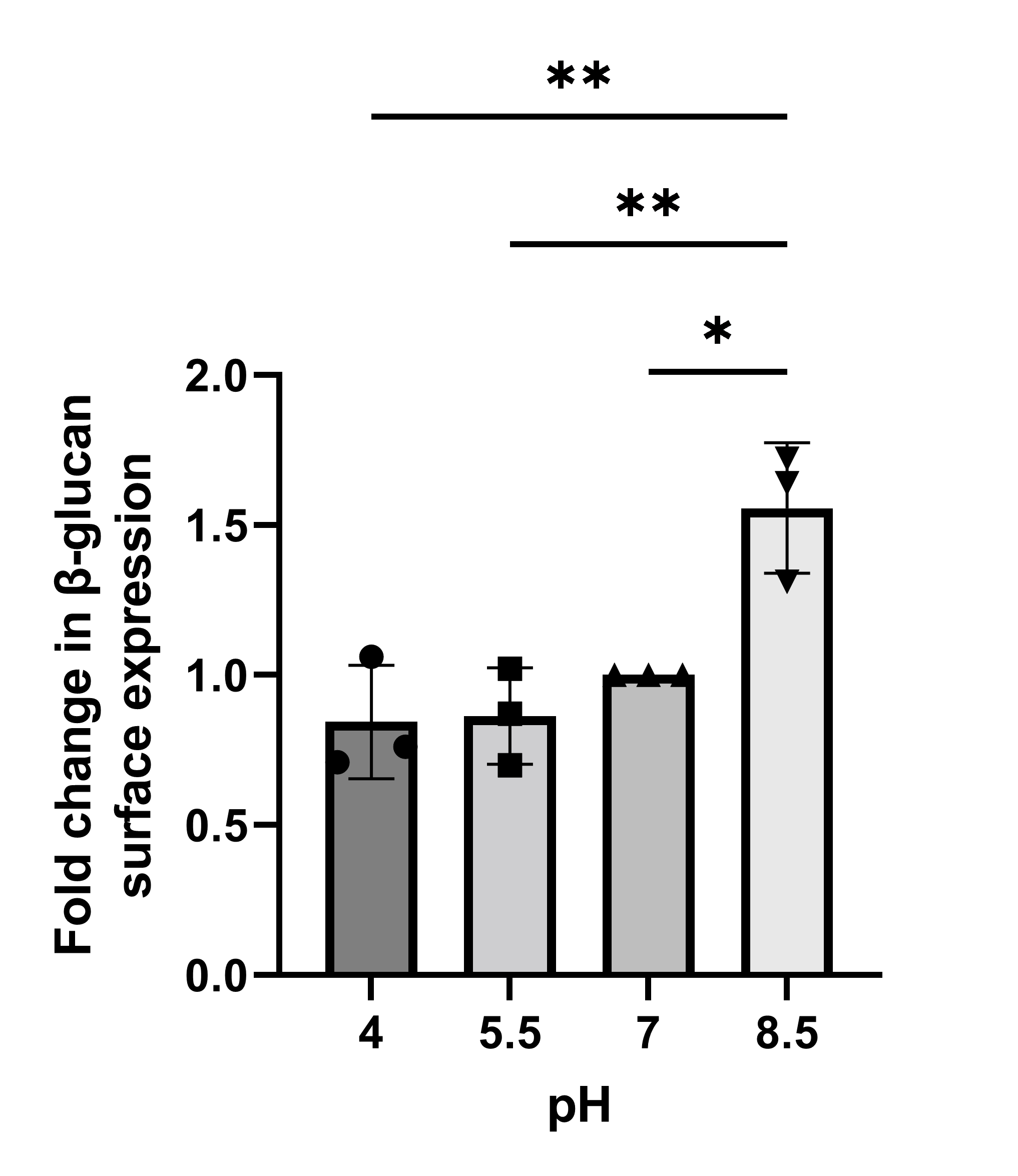

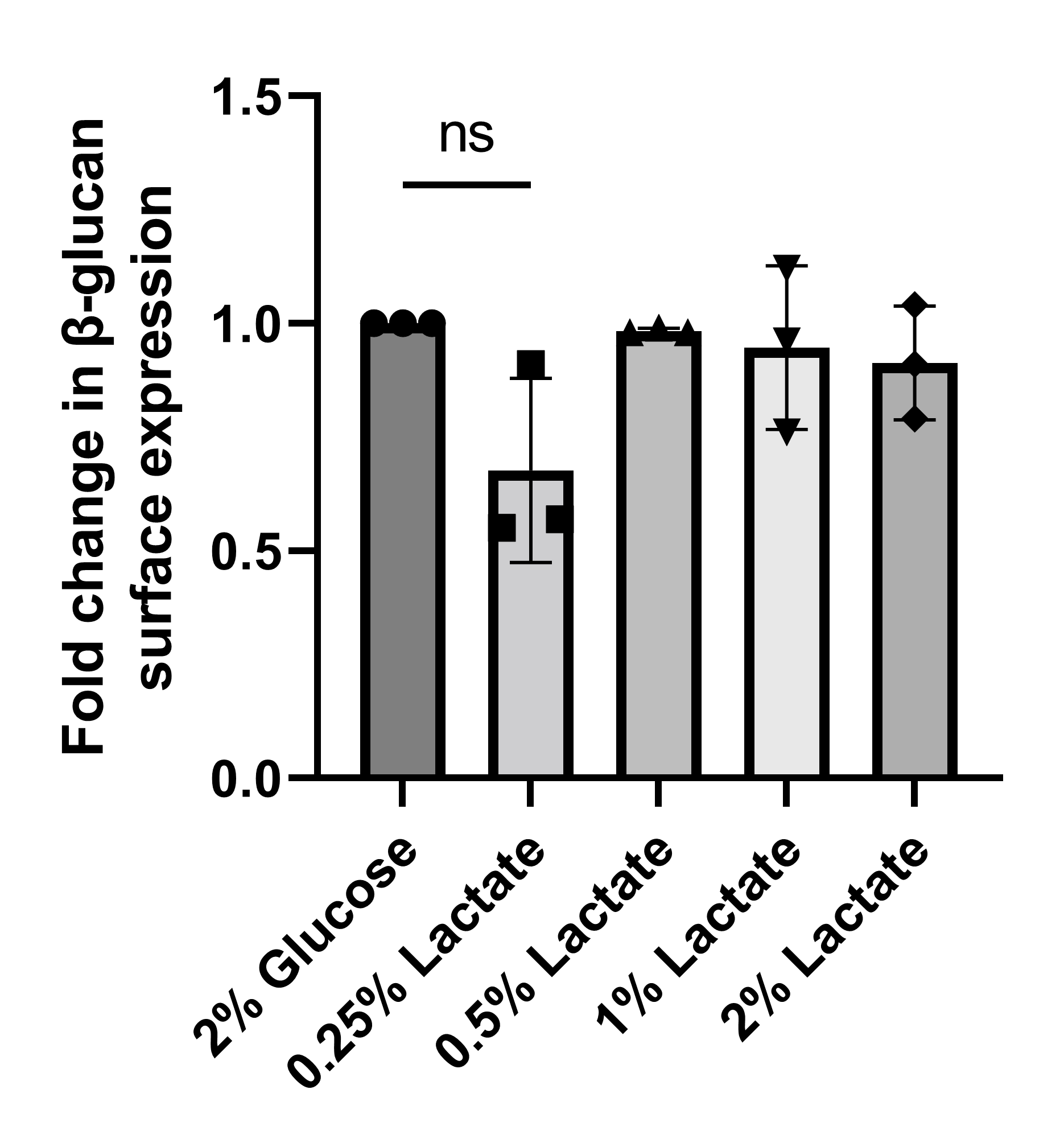

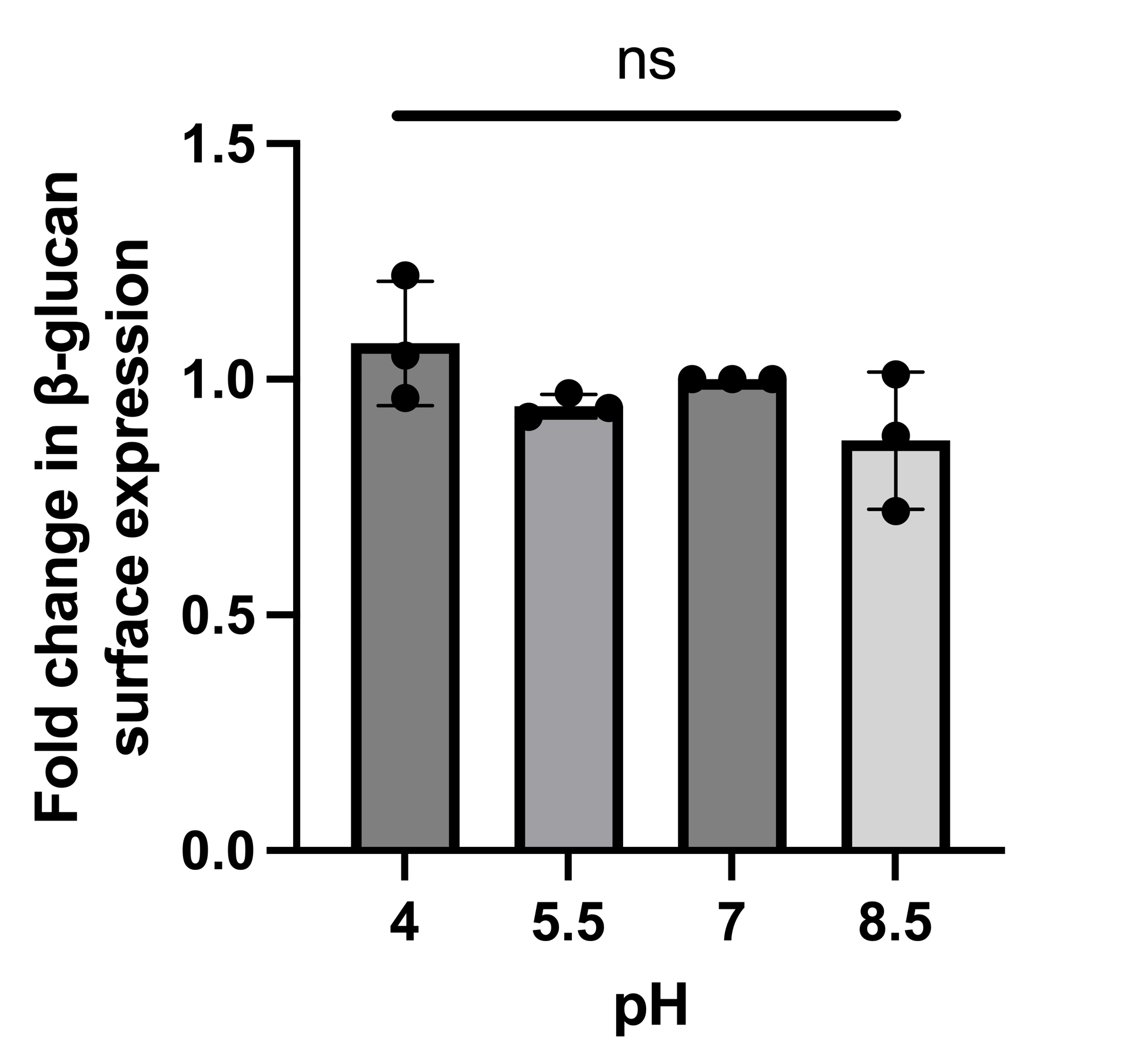

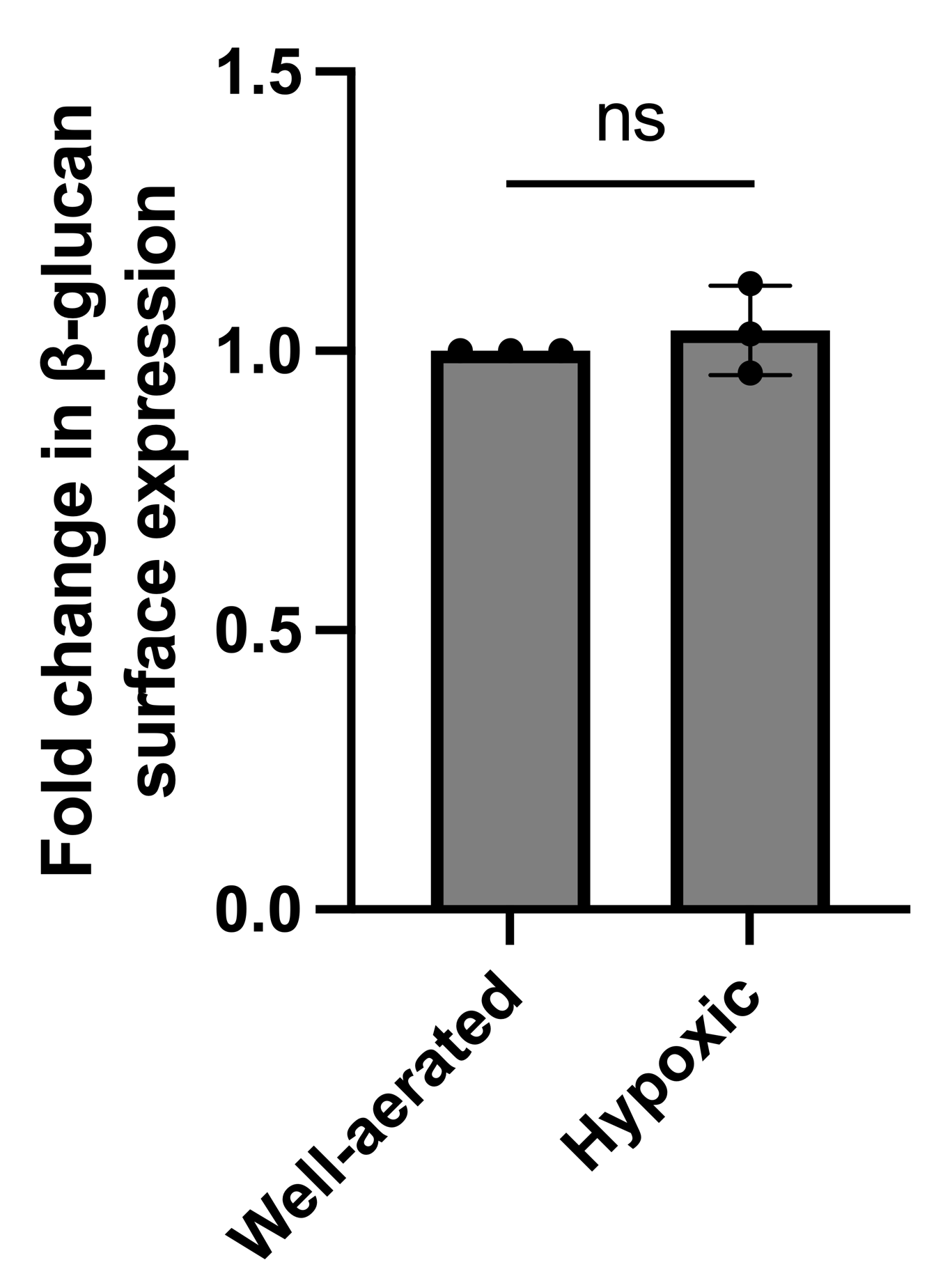


**Fig. S1** Culture condition-induced changes in *C. auris* β-glucan is strain-specific. Effect of lactate (**A**), hypoxia (**B**), and pH (**C**) on exposure levels of β-glucan in type strains of *C. auris* after a 5-h incubation at 37ºC. Bar graphs show the mean and SD from three independent biological replicates analyzed through paired t-test (B) or one-way ANOVA with Tukey’s multiple comparisons test (A, C). * *P* < 0.05, *** P* < 0.01, ns = not significant. The effect of antifungals was not experimented as both strains do not show multidrug resistance

**MYA-5002 (Clade III)**

**MYA-5001 (Clade II)**

***C. auris* ATCC:**

**a.**

**b.**

**c.**


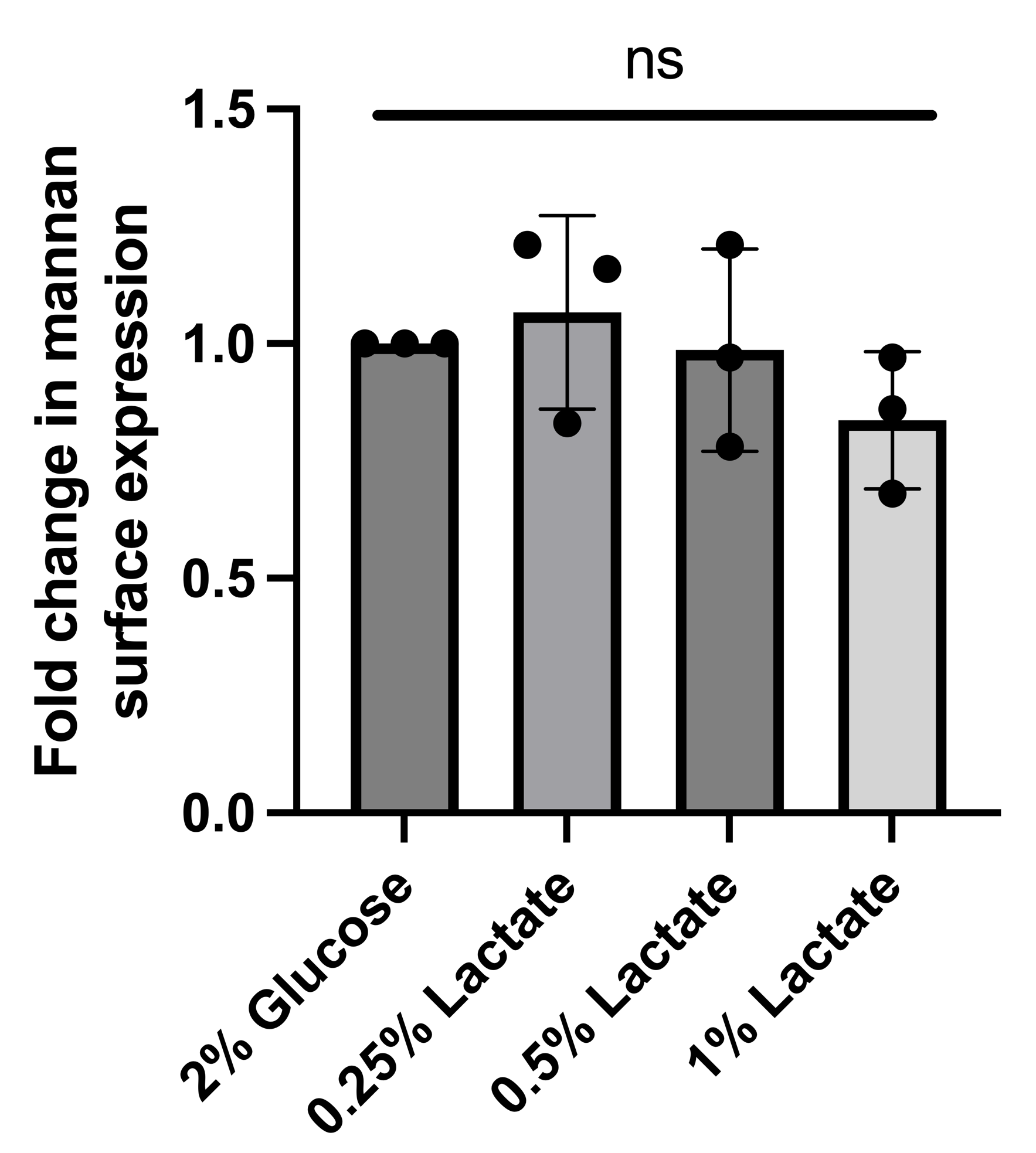

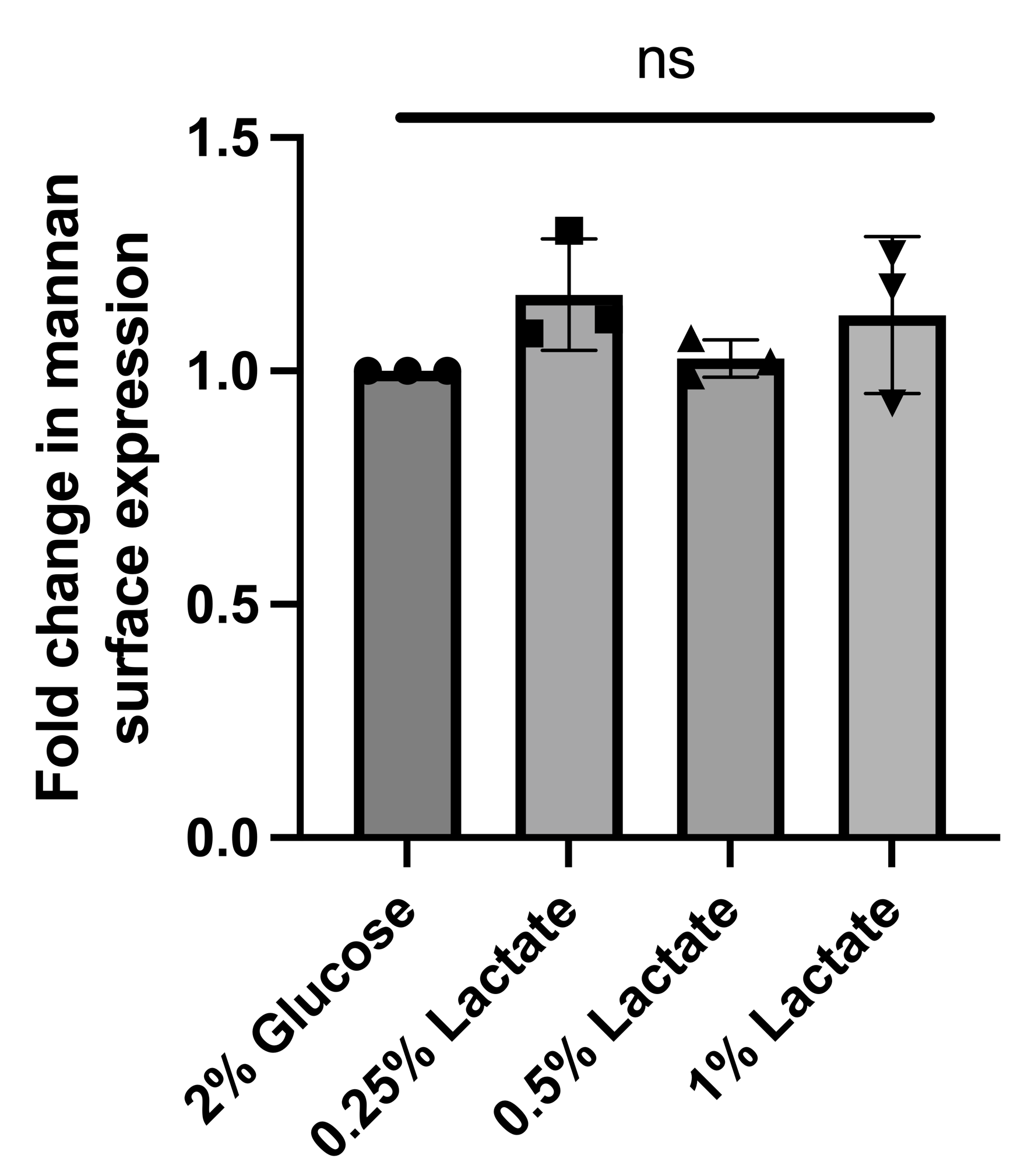

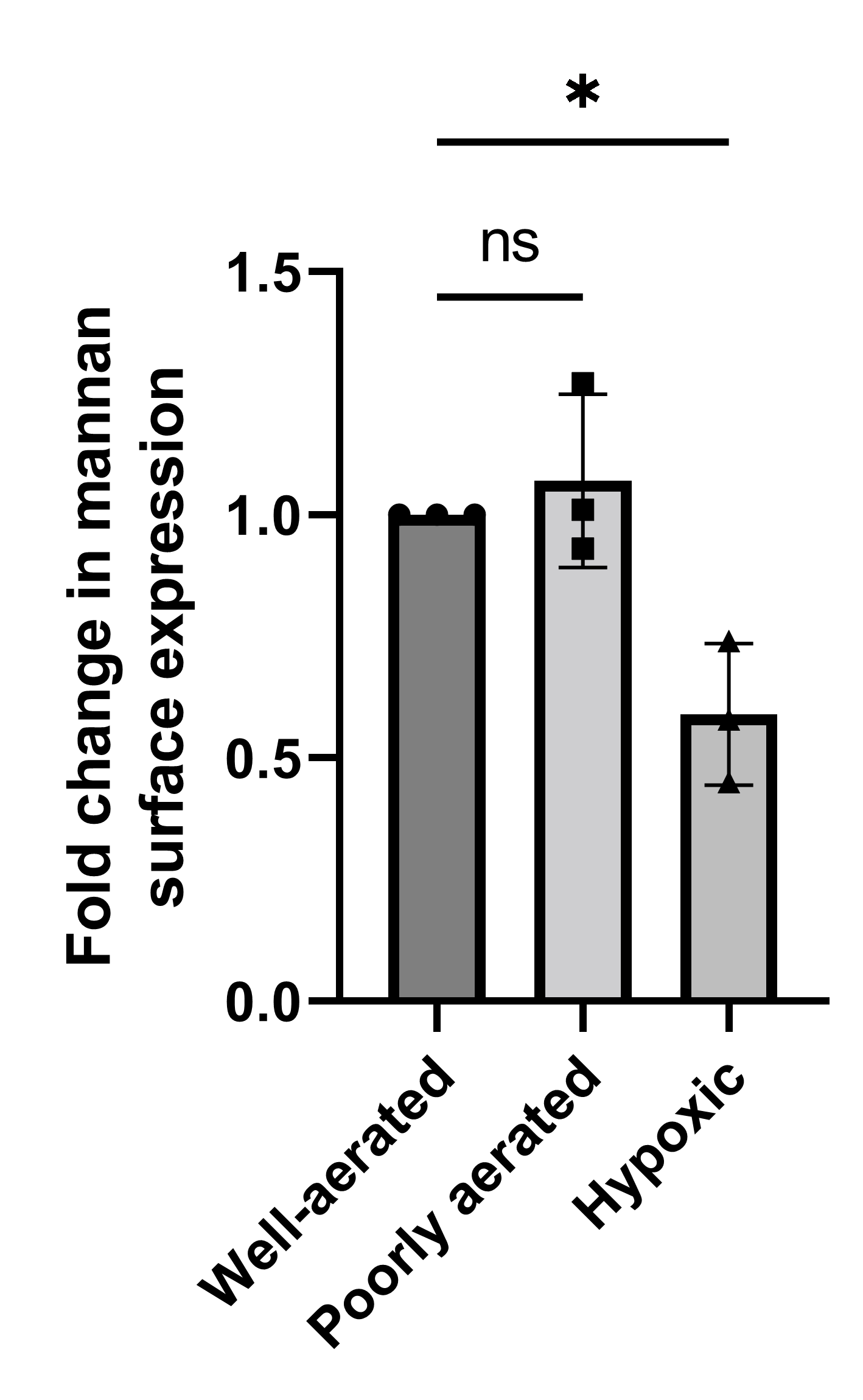

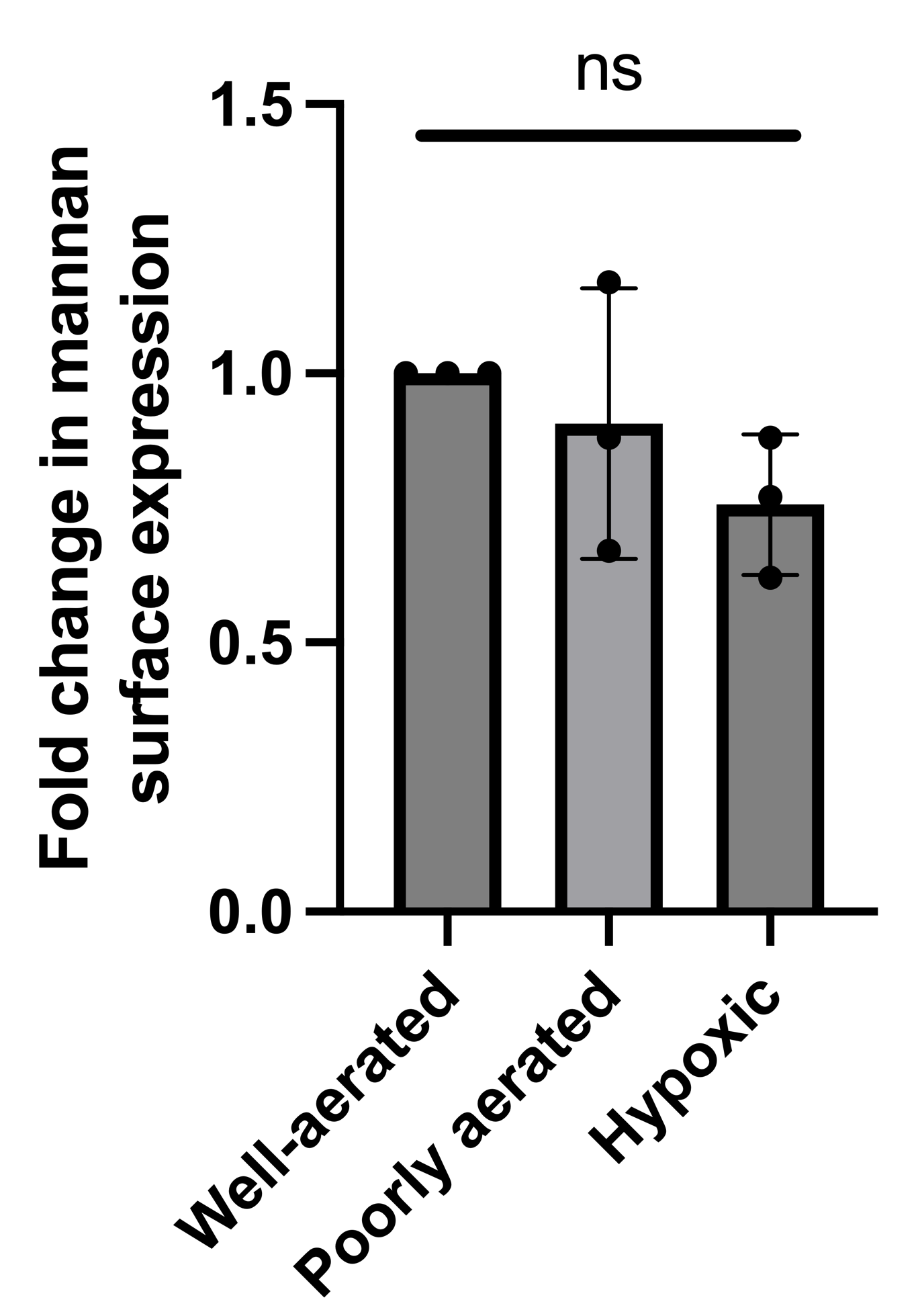

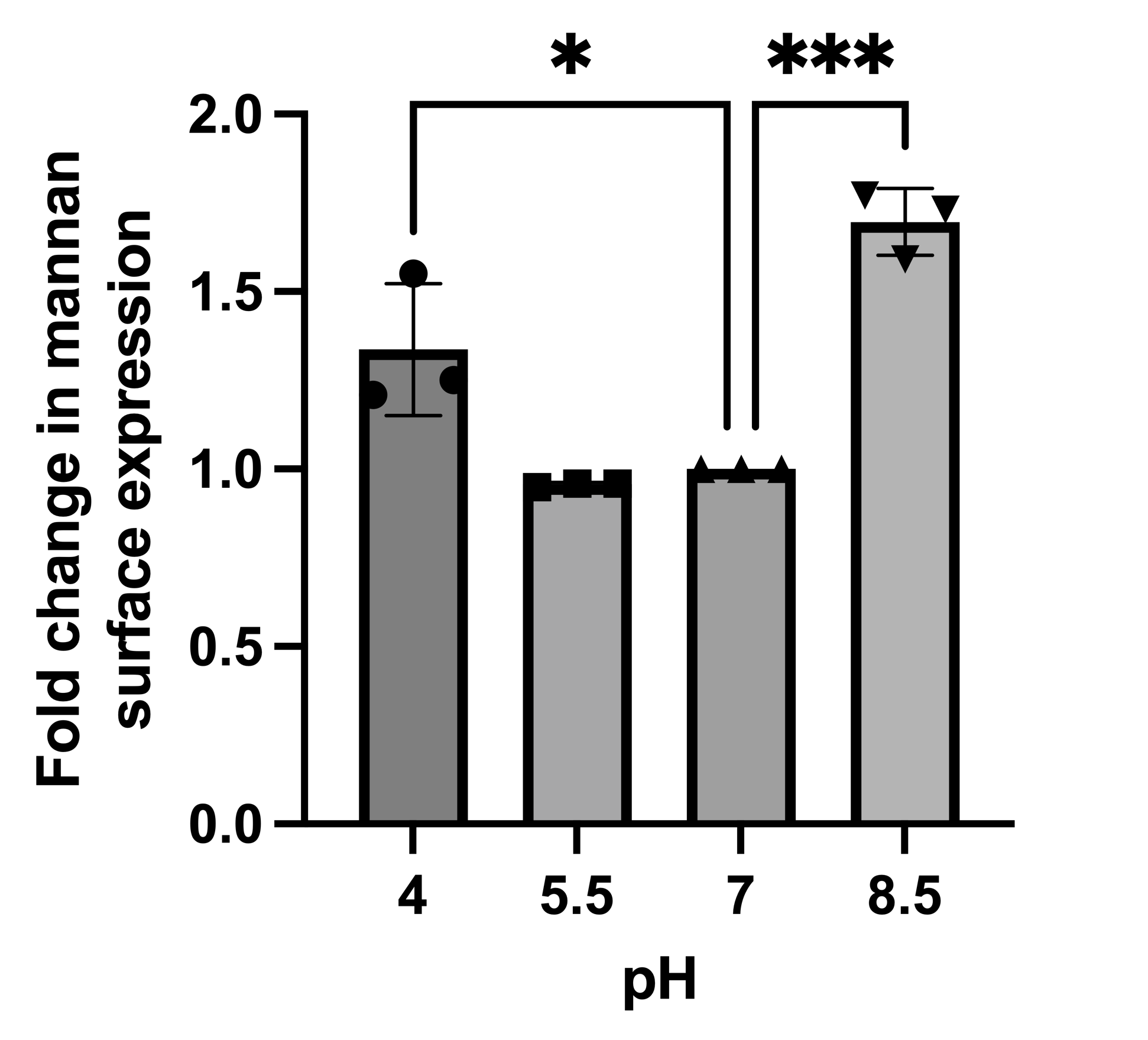

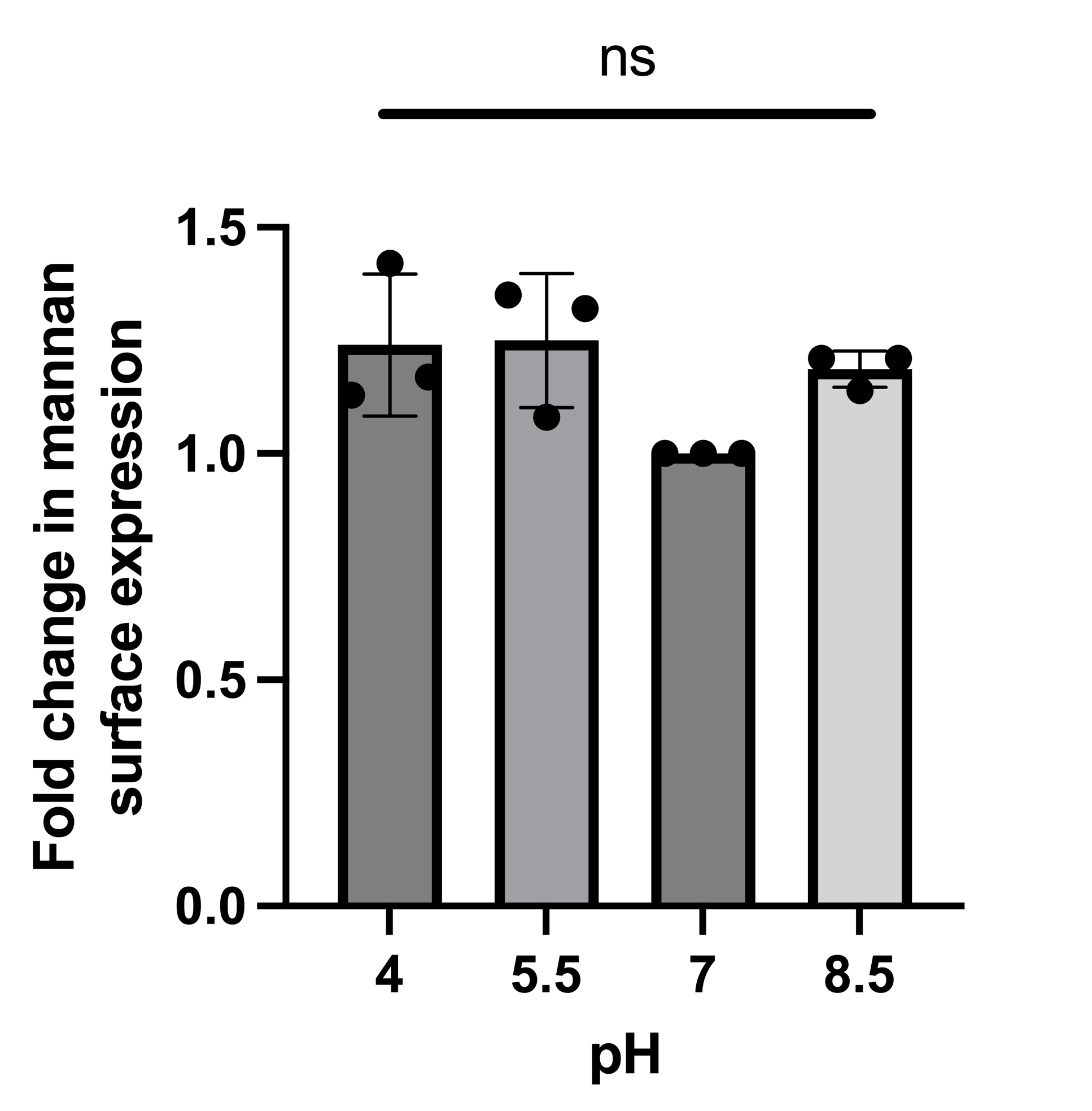


**Fig. S2** Culture condition-induced changes in *C. auris* mannan is strain-specific. Effect of lactate (**A**), low oxygen (**B**), and pH (**C**) on exposure levels of mannan in type strains of *C. auris*. Bar graphs show the mean and SD from three independent biological replicates analyzed through one-way ANOVA with Tukey’s multiple comparisons test. * *P* < 0.05, **** P* < 0.001, ns = not significant

Table S1 Median fluorescence intensities* of *Candida auris* UI001 grown in varying culture conditions and stained with Fc-hDectin-1a and Alexa Fluor 488-conjugated anti-human IgG_1_ Fc_γ_ fragment specific. Cells were analyzed using EC800 Flow Cytometry Analyzer with 488-nm laser in combination with FL1 (525/50) bandpass filter

| **Effect of carbon source** | **R1** | **R2** | **R3** | **R4** | **R5** | **R6** | **R7** | **R8** | **R9** |
| --- | --- | --- | --- | --- | --- | --- | --- | --- | --- |
| 2% glucose (control) | 168.49 | 333.76 | 209.08 | 429.35 | 189.38 | 417.92 | 406.79 | 116.52 | 85.05 |
| 0.25% lactate | (-) | (-) | (-) | 291.64 | (-) | (-) | (-) | 76.35 | 52.80 |
| 0.5% lactate | (-) | (-) | (-) | 177.83 | 64.36 | 228.76 | (-) | (-) | (-) |
| 1% lactate | 49.58 | 124.04 | 60.43 | (-) | (-) | (-) | (-) | (-) | (-) |
| 2% lactate | 106.50 | (-) | (-) | (-) | (-) | (-) | (-) | 53.28 | 50.03 |
| 1% glycerol | 156.79 | 291.64 | 187.69 | (-) | (-) | (-) | (-) | (-) | (-) |
| 2% glycerol | (-) | (-) | (-) | (-) | 186.01 | 465.55 | 417.92 | (-) | (-) |
| **Effect of oxygen** |  |  |  |  |  |  |  |  |  |
| Well-aerated (control) | 417.92 | 122.98 | 122.98 | 203.51 |  |  |  |  |  |
| Poorly aerated | 361.9 | (-) | 80.58 | 130.97 |  |  |  |  |  |
| Hypoxic | (-) | 77.04 | 63.78 | 127.49 |  |  |  |  |  |
| **Effect of temperature** |  |  |  |  |  |  |  |  |  |
| 30°C | 230.02 | 399.54 | 207.21 |  |  |  |  |  |  |
| 37°C | 271.39 | 375.16 | 168.49 |  |  |  |  |  |  |
| **Effect of pH** |  |  |  |  |  |  |  |  |  |
| 4 | 259.46 | 103.66 | 94.75 |  |  |  |  |  |  |
| 5.5 | 98.22 | 42.17 | 39.24 |  |  |  |  |  |  |
| 7 (control) | 33.98 | 19.28 | 16.85 |  |  |  |  |  |  |
| 8.5 | 137.00 | 27.14 | 28.39 |  |  |  |  |  |  |
| **Effect of antifungals** |  |  |  |  |  |  |  |  |  |
| No fluconazole (control) | 74.99 | 142.02 | 168.49 | 355.45 |  |  |  |  |  |
| 2 μg/mL fluconazole | 92.22 | 159.63 | 155.38 | (-) |  |  |  |  |  |
| 4 μg/mL fluconazole | 100.00 | 161.08 | 147.22 | (-) |  |  |  |  |  |
| 16 μg/mL fluconazole | 14.25 | 38.89 | (-) | 64.36 |  |  |  |  |  |

R = replicate

(-) = no samples for that particular replicate

*fluorescence intensity of samples fluctuates depending on how long the antibodies and fluorochrome are kept in the refrigerator

fold change values are used in statistical analysis; fold change = MFI of β-glucan::Alexa Fluor 488 in the experimental variable divided by the MFI of the

control

Table S1 Median fluorescence intensities* of *Candida auris* UI001 grown in varying culture conditions and stained with Fc-hDectin-1a and Alexa Fluor 488-conjugated anti-human IgG_1_ Fc_γ_ fragment specific. Cells were analyzed using EC800 Flow Cytometry Analyzer with 488-nm laser in combination with FL1 (525/50) bandpass filter (continuation)

| **Effect of antifungals** | **R1** | **R2** | **R3** | **R4** | **R5** |
| --- | --- | --- | --- | --- | --- |
| No micafungin (control) | 441.09 | 93.06 | 697.83 | 259.46 |  |
| 0.63 μg/mL micafungin | 278.81 | 54.25 | 361.90 | (-) |  |
| 1.25 μg/mL micafungin | 283.87 | 55.23 | (-) | 209.08 |  |
| 1.50 μg/mL micafungin | 289.03 | 42.55 | 410.47 | (-) |  |
| 2.50 μg/mL micafungin | 305.05 | 48.26 | 294.27 | (-) |  |
| 5 μg/mL micafungin | 224.68 | 46.98 | 310.59 | (-) |  |
| 10 μg/mL micafungin | 248.05 | 41.05 | 381.97 | (-) |  |
| 20 μg/mL micafungin | 254.83 | 57.25 | 441.09 | (-) |  |
| 40 μg/mL micafungin | 266.55 | 58.29 | 441.09 | (-) |  |
|  |  |  |  |  |  |
| No amphotericin B (control) | 406.79 | 73.65 | 264.16 | 122.98 | 93.06 |
| 31 ng/mL amphotericin B | (-) | 79.15 | 239.28 | 105.54 | (-) |
| 47 ng/mL amphotericin B | (-) | 80.58 | 216.74 | (-) | 93.90 |
| 63 ng/mL amphotericin B | 417.92 | 69.16 | 232.91 | (-) | (-) |
| 94 ng/mL amphotericin B | (-) | 79.86 | 224.68 | 115.48 | (-) |
| 125 ng/mL amphotericin B | 321.97 | 71.69 | 212.88 | (-) | (-) |
| 188 ng/mL amphotericin B | (-) | 73.65 | 232.91 | 100.00 | (-) |
|  |  |  |  |  |  |
| No 5-fluorocytosine (control) | 406.79 | 73.65 | 264.16 |  |  |
| 31 ng/mL amphotericin B | 333.76 | 59.89 | 212.88 |  |  |
| 63 ng/mL amphotericin B | 327.81 | 58.82 | 237.14 |  |  |
| 125 ng/mL amphotericin B | 352.27 | 63.21 | 214.80 |  |  |
| 150 ng/mL amphotericin B | 378.55 | 53.28 | 232.91 |  |  |
| 250 ng/mL amphotericin B | 307.81 | 48.70 | 209.08 |  |  |
| 500 ng/mL amphotericin B | 358.66 | 47.4 | 191.10 |  |  |

R = replicate

(-) = no samples for that particular replicate

*fluorescence intensity of samples fluctuates depending on how long the antibodies and fluorochrome are kept in the refrigerator

fold change values are used in statistical analysis; fold change = MFI of β-glucan::Alexa Fluor 488 in the experimental variable divided by the MFI of the control
